# Structural flexibility in the ordered domain of the dengue virus strain 2 capsid protein is critical for chaperoning viral RNA replication

**DOI:** 10.1007/s00018-025-05712-x

**Published:** 2025-04-28

**Authors:** Kamal K. Sharma, Palur Venkata Raghuvamsi, Daniel Y. K. Aik, Jan K. Marzinek, Peter J. Bond, Thorsten Wohland

**Affiliations:** 1 Centre for Bioimaging Sciences, National University of Singapore, 14 Science Drive 4, Singapore, 117557 Singapore; 2 Department of Biological Sciences, National University of Singapore, 14 Science Drive 4, Singapore, 117543 Singapore; 3 Bioinformatics Institute (BII), Agency for Science, Technology and Research (A*STAR), 30 Biopolis Street, #07-01 Matrix, Singapore, 138671 Republic of Singapore; 4 Department of Chemistry, National University of Singapore, 3 Science Drive 3, Singapore, 117543 Singapore

**Keywords:** RNA chaperone, RNA annealing and Strand displacement, Single molecule FRET, RNA molecular simulations

## Abstract

Viral replication necessitates intricate nucleic acid rearrangements, including annealing and strand displacement to achieve the viral RNA functional structure. Often a single RNA chaperone performs these seemingly incompatible functions. This raises the question of what structural and dynamic features of such chaperones govern distinct RNA rearrangements. While cationic intrinsically disordered regions promote annealing by playing a charge-screening role, how the same chaperone mediates strand displacement remains elusive. Here, we investigate the annealing and strand displacement of the 5’ upstream AUG region (5UAR) as chaperoned by the Dengue virus strain 2 capsid protein (Denv2C) as a model RNA chaperone. Through single molecule analysis and molecular simulations, we demonstrate that Denv2C regulates nucleic acid melting, folding, annealing, and strand displacement via flexibility in its ordered region. A mutation that renders the Denv2C ordered region rigid, converts Denv2C into a mere annealer. Our findings underscore the role of Denv2C’s disordered region as a “macromolecular counterion” during RNA annealing, while a flexible ordered region is crucial for effective strand displacement.

**Supplementary Information:**

The online version contains supplementary material available at 10.1007/s00018-025-05712-x.

## Introduction

**Fig. 1:**
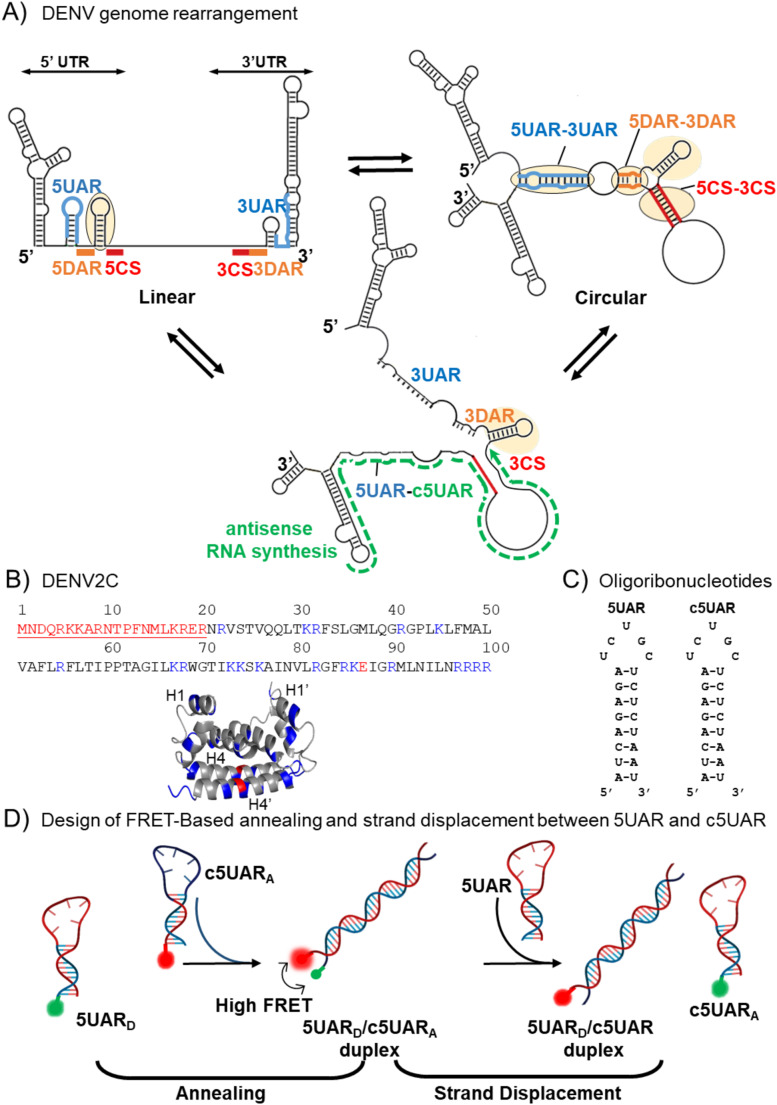
Schematic representation of DENV genome, Denv2C, essential RNA element sequences and FRET-based annealing and strand displacement approach used in this study. (**A**) Switch between two conformations of dengue genome during 5′-3′ panhandle formation. The 5′ and 3′ UTR region of dengue genome contain conserved sequences: 5CS and 3CS (red blocks), 5DAR and 3DAR (orange blocks), and 5UAR and 3UAR (blue lines). Annealing of 5UAR/c5UAR takes place during the (+)/(-) double stranded vRNA formation while the removal of antisense dengue RNA (green arrow) during linearization leads to strand displacement of 5UAR. (**B**) Denv2C amino acid sequence and structure (PDB: 1R6R). The protein is a homodimer with both basic (blue) and acidic (red) residues. The first 21 residues in the monomeric unit of Denv2C constitute the N-terminus disordered region (underlined) while rest of the residues (22–100 amino acids) constitute the ordered region. The α1 helix (H1) and α4 helix (H4) are labelled for both subunits of the protein, with H1 and H4 from one subunit and H1ʹ and H4ʹ from the other subunit. In the protein sequence of Denv2C monomer, residues in red are part of the N-terminus disordered region which are not shown in the structure. (**C**) Oligoribonucleotides. 5UAR sequence is derived from the 5′UTR regions of the genome. Their secondary structures were predicted using the mfold webtool (http://unafold.rna.albany.edu/). 5UAR is labelled with Cy3 as donor at the 5′ end while c5UAR is labelled with Atto647N at the 3’ end, respectively. (**D**) Schematic representation of the FRET-Based approach. The annealing of 5UAR_D_-c5UAR_A_ will lead to the formation of a duplex that is labelled with both Cy3 (green) and Atto647N (red). Due to the close proximity of donor and acceptor dyes, a high FRET efficiency and a decrease in the Cy3 intensity is observed. The addition of 5UAR during the strand displacement reaction led to the displacement of 5UAR_D_, which shifts the two dyes apart from each other, resulting in a decrease in FRET efficiency and the restoring of Cy3 fluorescence

RNA viruses use conserved RNA structures to regulate genome replication and interact with host ribosomes. Dengue virus (DENV) also relies on conserved cis-acting RNA elements, like the 5’ upstream AUG region (5UAR) [1, 2], 5′ cyclization sequence (5CS) [3], downstream AUG region (DAR) [4], capsid-coding hairpin (cHP) [5], downstream of 5′ cyclization sequence-pseudoknot (DCS-PK) [6] and pseudoknot (PK) [7] (Fig. 1A), to control essential replicative steps (Fig. 1A). DENV RNA synthesis involves genome cyclization (Fig. 1A), which is mediated either by protein-nucleic acid interactions or by long-range RNA-RNA interactions between 5’ and 3’ untranslated regions (UTRs) [1, 8]. This brings the RNA ends into close proximity [2, 8, 9], enabling synthesis of the intermediate minus-strand RNA [10, 11], that serves as a template for amplifying the (+) RNA strand to form new virions [2]. Therefore, this conformational switch of DENV (+) RNA between its circular and linear forms is crucial [2]. Along with the abovementioned RNA elements, the 21-nucleotide long 5UAR element in the 5’UTR plays a critical role by annealing to its complementary (-) RNA sequence (c5UAR) during minus-strand synthesis and displacing it during genome cyclization (Fig. 1A) [5]. The unwinding and displacement of the RNA structures allow the RNA-dependent RNA polymerase (NS5-RdRp) to synthesize new RNA strands. Earlier studies demonstrated that the DENV strain 2-capsid protein (Denv2C) modulates the annealing and melting of the 5UAR through its chaperone function, facilitating genomic rearrangements [12]. However, the mechanism by which Denv2C aids strand displacement of 5UAR remains unclear.

Denv2C, a highly basic protein, exists as a homodimer composed of two 100 amino acid subunits (Fig. 1B). Denv2C’s N-terminus (~ 1–24 amino acids) features a flexible and disordered region, while the core ordered region (~ 24–100 amino acids) exhibits an asymmetric charge distribution. One side of the dimer surfaces contains hydrophobic patches contributed by the α2 helices, while the opposite side is enriched with positively charged residues from the α4 helices (Fig. 1B). This structural duality allows Denv2C to interact with the viral lipid membrane via its hydrophobic regions and the negatively charged RNA genome through its positively charged helices on the other side [13].

As a member of the RNA chaperone family, Denv2C resolves kinetically trapped misfolded RNAs, guiding them towards compact, thermodynamically stable and biologically active conformations via its chaperone function [14–22]. Comparison across RNA chaperones [22–37] reveals minimal structural similarities, yet many, like Denv2C, are enriched in positively charged and polar residues and exhibit intrinsically disordered regions (IDRs). These IDRs enhance nucleic acid folding by providing local charge screening [38]. Although only 54% of RNA chaperones [39] and 36.7% of protein chaperones [39] possess IDRs, the contribution of structured regions to chaperone functionality cannot be excluded. For example, HIV-1 RNA chaperones Tat and NCp7 demonstrate differing chaperone abilities due to their structural domains. The folded zinc fingers in HIV-1 NCp7 transiently destabilize nucleic acids and modify nucleobase dynamics, underscoring the critical role of ordered regions [40].

These findings raise several questions: (i) Are IDRs sufficient for chaperone activity? (ii) How do the structured and ordered regions influence RNA chaperone function? Here, we employed single-molecule fluorescence and molecular dynamics (MD) simulations to explore two key aspects of Denv2C function: (i) its role during the annealing and strand displacement kinetics of the 5UAR to its complementary sequence (Fig. 1B) during vRNA genome rearrangement [1, 8, 12, 41] (Fig. 1A); and (ii) how the ordered region (~ 24–100 amino acids) influences its chaperone function.

To dissect these functions, we substituted the conserved serine residues in Denv2C with less-polar Cysteine residues at (i) the 34th position (Denv2C^S34C^) in Denv2C’s ordered region and (ii) the 24th position (Denv2C^S24C^) in the protein’s disordered region (Figure S1A). While both Ser24 and Ser34 are conserved across dengue serotypes, replacing Ser34 resulted in resistance to ST-148, a known capsid inhibitor [42, 43]. ST-148 interacts with the DenvC dimer at a cleft formed by the α1 helix of one dimer (involving Ser34) and α1 and α3 helices of the other dimer, inducing structural changes in the ordered region [42, 43]. Ser34 is critical for maintaining the structural integrity of Denv2C by forming hydrogen bonds with neighboring Gly36 and Met37, which stabilize the local protein scaffold [42, 43]. Its regulation of Phe33, a residue integral to rapid protein folding and scaffold stability due to its hydrophobic nature, further underscores its importance [44]. These findings highlight Ser34 as a key determinant of Denv2C functionality, linking structural dynamics to its role in vRNA rearrangement and resistance to capsid inhibitors. Conversely, Ser24, located in the disordered region, is likely to be less critical for protein folding and scaffold stability.

We investigated the role of Denv2C and its ordered region during viral RNA rearrangement by studying the annealing and strand displacement kinetics of the 5UAR/c5UAR duplex using Förster Resonance Energy Transfer (FRET). The 5UAR was labelled with Cyanine3 (Cy3) as the donor fluorophore at the 5’ end, while c5UAR was labelled with Atto 647N as the acceptor at the 3’ end (Fig. 1C). Annealing increased donor-acceptor proximity, quenching donor fluorescence, whereas strand displacement, induced by excess non-labelled 5UAR, restored donor fluorescence by separating the dyes (Fig. 1C). Combining single-molecule FRET (smFRET) and molecular simulations, we further explored RNA folding dynamics by examining how Denv2C and its ordered region impact the kinetics of nucleic acid folding and unfolding, using a stem-loop hairpin (HP) as a known model [38] (Figure S2).

As a prototypical RNA chaperone from enveloped viruses, elucidating Denv2C’s function and the structural determinants underlying its role in promoting nucleic acid transitions provides critical insights into viral replication mechanisms and paves the way for developing targeted antiviral strategies against emerging pathogens.

## Materials and methods

### Oligonucleotides

All oligonucleotides (RNAs (ORN) and DNAs (ODN)) were synthesized by Integrated DNA Technologies (Singapore). The donor-labelled essential RNA element 5UAR was synthesized with cyanine3 (Cy3) at the 5’ end (Fig. 1C). The acceptor-labelled complementary RNA element c5UAR were synthesized with Atto647N at the 3’ end (Fig. 1C). The 2-AP ORN sequences were synthesized by replacing adenine nucleoside at either the 6th, or 11th or 20th position in the 5UAR hairpin by 2-amino purine (Figure S3). The doubly labelled RNA based hairpin (HPr) was synthesized with Atto647N, cyanine3 and biotin at the 5’end, at the 48th position towards 3’ end and at the 3’ end, respectively (Figure S2). The DNA based hairpin (HPd) had a sequence like the RNA HP with similar labelling modifications (Figure S2). All oligonucleotides were purified by the manufacturer using HPLC.

### Denv2C protein and its mutant synthesis and purification

Denv2C protein and its mutants were expressed and purified as described in an earlier study [12], in the presence of dithiothreitol (DTT) to avoid any disulfide bond formation between cysteine residues. The obtained A_260_/A_280_ values of ~ 0.7 for the purified proteins were found close to the theoretical value of 0.57 for a protein sample not contaminated by nucleic acids.

### Fluorescence spectroscopy

Fluorescence spectroscopy measurements were done on a Cary Eclipse Fluorescence Spectrophotometer (Agilent) with a temperature control module, using Hellma^®^ fluorescence cuvettes with an internal chamber volume of 50 µL. Excitation and emission wavelengths of 532 nm and 560 nm were used to track the intensity of Cy3 in real-time. Annealing reactions were performed in second order conditions by adding acceptor-labelled ORN to donor-labelled ORN with a starting 1:1 ratio while the strand displacement reactions were performed in pseudo first order conditions with the concentration of non-labelled ORN being at least 10-fold more than the doubly labelled annealed complexes. Equal volumes of both reactants were mixed at the start of the reaction to prevent high local concentrations of either reactant. A final concentration of 2 µM protein was used for monitoring the effect of chaperone on strand displacement reactions while a 4-times concentration of protein, compared to total used ORN concentration, was used for monitoring annealing reactions. All annealing reactions were performed in 50 mM HEPES, 150 mM NaCl, 1 mM MgCl_2_, pH 7.5 buffer at 20˚C while strand displacement reactions were performed in same buffer conditions at 37˚C. All curve fitting was done using OriginProTM software (ver 9.55).

### Total-internal reflection fluorescence (TIRF) microscopy

For imaging, we employed a modified version of the alternating-laser excitation (ALEX) approach [45, 46]. Instead of using a high-speed digital signal generator to synchronize illumination and detection, we opted to use the camera’s TTL FIRE output to trigger either the first or the second laser at a pre-defined on-time when a new frame is exposed. The TIRF microscope setup featured an objective-based TIRF microscope equipped with 100× oil immersion (PlanApo; 100×, NA 1.45; Olympus) objective. Our setup included laser lines 532 nm LS 150 mW (OBIS Coherent, CA) and 638 nm LuxX 200 mW (Omicron, Germany) with modulation capabilities of up to 150 kHz, both combined into a single fiber output using a laser engine (Omicron LightHUB+, Germany). Divergent excitation lights were coupled through a two-lens TIRF illuminator module IX2-RFAEVA-2 (Olympus, Japan) before passing through a dichroic (Di01-R405/488/532/638, Semrock, USA) focusing light to the back focal plane of the TIRF objective. The fluorescence emission passes through the same dichroic mirror for the dual-channel measurements. It is further separated with an image splitter device to split spatially equivalent emission signals into two halves of a camera chip (OptoSplit II, Cairn Research, UK). The image splitter was fitted with 640 nm dichroic, 680/42 (Semrock, USA), and ET575/50m (Chroma Technology Corp, USA) emission filters. We used a back-illuminated EMCCD camera (iXON 860; 128 × 128 pixels; Andor, UK) for the detection. Finally, the image stack was collected with a home-built data acquisition software running directly through Fiji built using Andor Software Development Kit (SDK) (version: 2.103.30031.0).

### SmFRET experiments

Biotin labelled hairpins (HPr and HPd) were immobilized on PEGylated coverslips through a streptavidin–biotin interaction. All samples were measured in flow channels formed by double-sided sticky tapes. Images of immobilized HPs were taken at a rate of 333 frames per second, using excitation powers of 40 mW (367 W/cm^2^) (638 nm) and 37 mW (340 W/cm^2^) (532 nm) measured before the objective. Imaging was performed in a buffer containing 50 mM HEPES (pH 7.5), 150 mM NaCl, 1 mM MgCl_2_, 2 mM Trolox, 200U/mL glucose oxidase, 2000 U/mL catalase and 0.8% glucose. Trolox is a triplet state quencher, while glucose oxidase and catalase serve as an oxygen scavenger system; both prevent premature bleaching of the fluorophores. For each sample condition, at least 70–100 molecules showing transitions were observed from the recorded movies. Analysis of the acquired movies was performed using iSMS: single-molecule FRET microscopy software by Soren Preus et al. [47]. iSMS is an interactive toolkit for the comprehensive analysis of smFRET TIRF-microscopy data. iSMS integrate and automates common procedures in smFRET data analysis: molecule localization, intensity-trace integration, quantitative FRET determination, FRET distribution analysis, molecule subpopulation analysis and transition state dynamics analysis.

### Modeling of Denv2C protein, RNA, and complexes

The full-length dengue Denv2C protein dimer (PDB ID: 1R6R [13] was modelled as described previously [48]. In silico mutation of S34C (Denv2C^S34C^) was performed using the CHARMM-GUI pdb-reader module [49]. Default protonation states at neutral pH were assigned to ionizable residues using the CHARMM36m force field( https://pmc.ncbi.nlm.nih.gov/articles/PMC5199616/). The RNA molecules i.e., HPr and 5UAR were modelled using simRNAweb [50]. In the case of Denv2C protein: RNA complexes, each RNA molecule was initially placed in close proximity to the α4-helix (5UAR) in three different orientations (Linear 5UAR and HPr). Models of the Denv2C protein: RNA complexes, Denv2C protein alone and RNA alone were solvated using the TIP3P [51] water model along with 150 mM of NaCl and 1 mM of Mg^2+^ according to the experimental setup. Each system was subjected to ≤10,000 steps of energy minimization using the steepest descent algorithm. The energy-minimized structures were further subjected to 125 ps of equilibration in the NPT ensemble (constant number of particles, pressure and temperature) with position restrained protein and RNA backbone atoms with a force constant of 400 kJ nm^− 2^. The Particle mesh Ewald (PME) method (ref: https://pubs.aip.org/aip/jcp/article-abstract/103/19/8577/180219/A-smooth-particle-mesh-Ewald-method?redirectedFrom=fulltext) was used to treat long-range electrostatic interactions with a real space cut-of 1.2 nm. A cutoff of 1 nm was used with a switching function for Lenard-Jones interactions. Production simulations were run for 500 ns in the NPT ensemble. The Nosé-Hoover thermostat (303 K) and Parrinello-Rahman barostat (1 bar) were used to maintain temperature and pressure respectively. Constraints were applied to covalent bonds involving hydrogens using the LINCS algorithm (ref: https://onlinelibrary.wiley.com/doi/abs/10.1002/(SICI)1096-987X(199709)18:12%3C1463::AID-JCC4%3E3.0.CO%3B2-H). Equations of motion were integrated using the leapfrog algorithm with a 2-fs time step.

All MD simulations were performed using GROMACS 2020.2 (ref: https://pubmed.ncbi.nlm.nih.gov/16211538/) and all simulation systems are detailed in Table S1. Simulation snapshots were generated using VMD [52], ChimeraX [53–55] and Pymol. The base pair, base step parameters and hydrogen-bonds (H-bonds) between base pairs of RNA were calculated using X3DNA tools [56]. Cluster analysis was performed on protein backbone atoms of the folded regions of the C protein (residues 20–100) using the Gromos method with an RMSD cutoff of 0.25 nm. A cutoff distance of 0.6 nm between atom pairs was used to calculate numbers of contacts. A contact frequency that ranges from 0 to 1, corresponding to the fraction of simulation time in which two atom pairs were within the cutoff distance, was calculated and plotted for Denv2C: RNA complex simulations. Gromacs analysis tools were used to measure other aspects of protein dynamics and protein: RNA interactions. All simulation parameters used for simulation setup are summarized in Table S1.

## Results and discussion

### S34C mutation in Denv2C transforms its function from an RNA chaperone to an RNA annealer

**Fig. 2:**
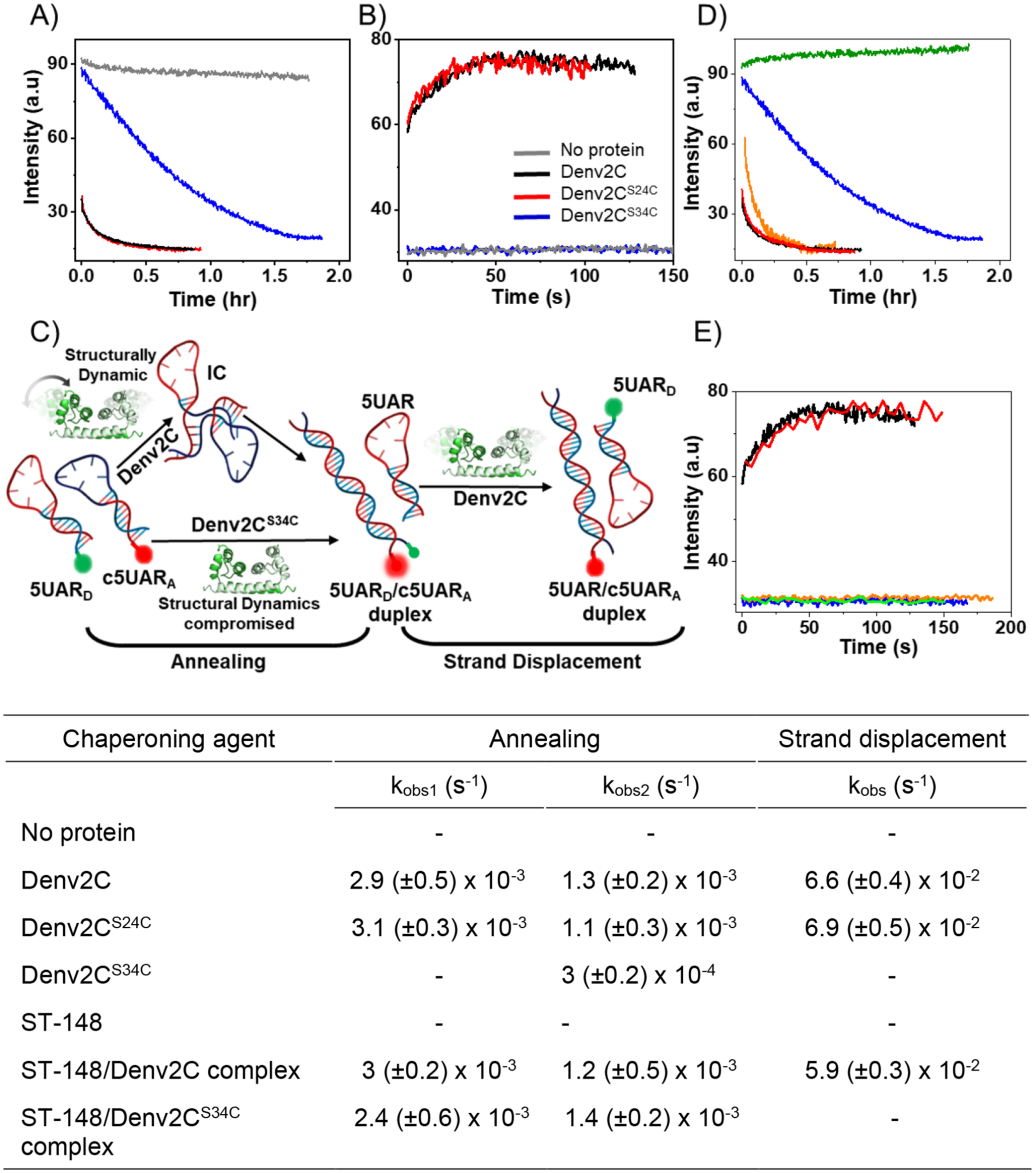
Impact of S34C mutation and ST-148 on the 5UAR/c5UAR annealing and strand displacement kinetics. (**A** and **B**) Progress curves of (**A**) annealing between 10 nM Cy3-labelled 5UAR with 100 nM Atto647N-labelled c5UAR and (**B**) strand displacement with1 µM non-labelled 5UAR from 20 nM preformed 5UAR/c5UAR duplex in the absence (gray trace) or in the presence of either 440 nM Denv2C (black trace) or 440 nM Denv2C^S34C^ (blue trace) or 440 nM Denv2C^S24C^. Annealing reactions were performed at 20˚C while strand displacement reactions were performed at 37˚C. Excitation and emission wavelengths were 532 nm and 560 nm, respectively. Equation (1) or Eq. (2) from supplementary part were used for fitting traces and obtained apparent rates are shown in the table (**C**) Schematic representation of the annealing and strand displacement reaction pathway. In the presence of Denv2C, the annealing of the donor labelled 5UAR (5UAR_D_) and acceptor labelled c5UAR (c5UAR_A_) propagates through a single two-step kinetic pathway where doubly labelled duplex (5UAR_D_/c5UAR_A_) is formed via a stem-stem intermediate complex (IC). A divergent and ~ 80-folds slower annealing takes place in the presence of structurally compromised Denv2C^S34C^. During strand displacement, Denv2C initiates and catalyzes the reaction by facilitating the displacement of donor-labelled 5UAR from the 5UAR_D_/c5UAR_A_ duplex in the presence of invading non-labelled 5UAR. In contrast, the structurally and dynamically compromised Denv2C^S34C^ fails to displace 5UAR, highlighting the critical role of structural integrity in this process. (**D** and **E**) Real time progress curves of (**D**) annealing between 10 nM Cy3-labelled 5UAR and 100 nM Atto647N-labelled c5UAR and (**E**) strand displacement with 1 µM non-labelled 5UAR from 20 nM 5UAR/c5UAR duplex in the presence of 8.8 µM ST-148 (green trace), 440 nM Denv2C (black trace), 440 nM Denv2C^S34C^ (blue trace), complex of 8.8 µM ST-148 + 440 nM Denv2C (red trace) and complex of 8.8 µM ST-148 + 440 nM Denv2C^S34C^ (orange trace). Annealing reactions were performed at 20˚C while strand displacement reactions were performed at 37˚C. Excitation and emission wavelengths were 532 nm and 560 nm, respectively. Equation (1) or Eq. (2) from supplementary part were used for fitting traces and obtained apparent rates are shown in the table

Initially, we explored the influence of Denv2C mutations at positions 24 (Denv2C^S24C^) and 34 (Denv2C^S34C^) on the annealing kinetics of Cy3-labelled 5UAR to its complementary Atto 647N-labelled c5UAR, as well as on the strand displacement of Cy3-labelled 5UAR from pre-formed Cy3/Atto 647N-labelled 5UAR/c5UAR duplexes (Fig. 2). We observed that the Cy3 fluorescence of donor-labelled 5UAR in the presence of Atto647N-labelled c5UAR during real time annealing kinetics shows a continuous decrease in FRET before reaching a plateau with the formation of the duplex that contains both Cy3 and Atto647N in proximity (Fig. 2A). As expected, the Denv2C-promoted 5UAR/c5UAR annealing reaction showed a rapid real-time Cy3 fluorescence decrease (Fig. 2A) and a drastic increase in the values of the kinetic parameter (Fig. 2 table) when compared to the absence of protein. A comparable effect was obtained during Denv2C^S24C^-promoted 5UAR/c5UAR annealing indicating that the mutation at the 24th position does not alter the annealing properties of Denv2C. However, Denv2C^S34C^-mediated 5UAR/c5UAR annealing was significantly slower compared to wild-type Denv2C, highlighting the detrimental impact of the mutation at the 34th position. These results indicate that the S34C mutation in Denv2C is probably able to alter structural properties of the Denv2C ordered region that may alter the chaperoning ability of the protein as detailed in the 5UAR/c5UAR annealing reaction mechanism (Fig. 2C and Supplementary part M1). A ~ 20-fold difference between values of k_diss_ suggest that the 5UAR/c5UAR duplexes formed during Denv2C^S34C^-promoted annealing have increased stability as compared to the duplexes formed during Denv2C-promoted annealing (Supplementary Table M1). In addition, the compromised flexibility in the Denv2C ordered region propagates Denv2C-promoted 5UAR/c5UAR annealing via a divergent reaction pathway (Fig. 2C and compare scheme 1 and 2 in Supplementary part M1).

During strand displacement reactions, the introduction of non-labelled 5UAR to pre-formed Cy3/Atto 647N-labelled 5UAR/c5UAR duplexes triggered a rapid increase in Cy3 fluorescence in the presence of both Denv2C and Denv2C^S24C^ (Fig. 2B). This finding suggests that the mutation at the 24th position of Denv2C does not compromise its chaperone properties, as both Denv2C and Denv2C^S24C^ demonstrate the ability to initiate strand displacement - a key characteristic of RNA chaperones [15, 17, 57, 58]. By contrast, we observed no changes in the Cy3 fluorescence of the Cy3/atto647N-labelled 5UAR/c5UAR duplexes in the presence of Denv2C^S34C^, suggesting that no strand displacement takes place (Fig. 2B). We tested a 100-fold molar excess of non-labelled 5UAR to assess strand displacement (Supplementary Figure M2), but even at these high concentrations of complementary strands, no increase in Cy3 fluorescence was observed. This confirms that Denv2C^S34C^ is unable to initiate strand displacement, as detailed in the 5UAR/c5UAR strand-displacement reaction mechanism (Fig. 2C and Supplementary part M2). Collectively, our results reveals that the S34C mutation compels Denv2C^S34C^ to function as an RNA annealer. RNA annealers accelerate annealing of complementary RNAs by binding to one or both RNAs. This may work by enhancing the effective local concentration with charge neutralization, thereby increasing the probability of RNA-RNA interactions.

### ST-148 confirms nucleic acid annealer characteristics of Denv2C^S34C^

ST-148, a potent dengue virus capsid inhibitor, enhances DenvC dimer self-interaction by promoting the formation of DenvC tetramers (i.e. dimers of dimers) [43]. Classified as a direct protein-protein interaction stabilizer, ST-148 induces hydrophobic and electrostatic interactions in a cleft between the capsid dimers. Specifically, residues Val_26_, Leu_29_, Arg_41_, and Arg_68_ of one dimer, along with Ser_34_ of the other dimer, form the binding site for ST-148 on DenvC [43]. While this inhibitor disrupts the structural rigidity of dengue nucleocapsids and inhibits the production of infectious dengue virus, it does not impact viral RNA replication [43]. Consequently, the presence of ST-148 is not expected to affect Denv2C-promoted annealing or strand displacement kinetics, which are critical for viral RNA replication.

To test this, we conducted 5UAR/c5UAR annealing (Fig. 2D) and strand displacement (Fig. 2E) reactions in the presence of pre-formed ST-148/Denv2C complex. As anticipated, the ST-148/Denv2C complex-promoted 5UAR/c5UAR annealing reaction showed similar real-time Cy3 fluorescence decrease (Fig. 2D) and comparable kinetic parameters (Fig. 2 table) when compared to Denv2C-promoted 5UAR/c5UAR annealing. Likewise, the ST-148/Denv2C complex-promoted 5UAR/c5UAR strand displacement reaction exhibited similar Cy3 fluorescence increase (Fig. 2E) and corresponding kinetic parameter (Fig. 2 table) to the Denv2C-promoted 5UAR/c5UAR strand displacement reaction. These findings demonstrate that ST-148 does not alters the annealing or strand displacement activities of Denv2C.

In contrast, the ST-148/Denv2C^S34C^ complex drastically accelerated 5UAR/c5UAR annealing as compared to Denv2C^S34C^ alone (compare the black and orange traces in Fig. 2D). Interestingly, the kinetic parameters for the ST-148/Denv2C^S34C^ complex-promoted 5UAR/c5UAR annealing were similar to those observed with Denv2C-promoted 5UAR/c5UAR annealing (Fig. 2 table), suggesting an increase in the local concentration of Denv2C^S34C^ molecules [43]. This localized concentration boost may facilitates efficient charge screening of nucleic acids [38], which is a fundamental mechanism for RNA annealing by an RNA annealer. Furthermore, the absence of 5UAR/c5UAR strand displacement activity in the ST-148/Denv2C^S34C^ complex (Fig. 2E) further reinforces the notion that Denv2C^S34C^ functions as an RNA annealer rather than as RNA chaperone.

Collectively, the results also indicate that Denv2C, in its dimeric form, acts as the essential and basic RNA chaperone unit for accelerating and initiating nucleic acid annealing and strand displacement reactions. Overall, our findings indicates that Denv2C^S34C^ may induce structural alterations in the RNA upon binding, enabling it to facilitate annealing while rendering it incapable of promoting strand displacement (Fig. 2C). The results are in line with earlier studies demonstrating the Denv2C’s ability to modulate the intrinsic dynamics as well as structural conformations of RNA hairpins [12].

### Denv2C interacts with and destabilizes the lower stem region of the 5UAR hairpin

To understand how Denv2C induces structural alterations in the RNA upon binding, we investigated the 5UAR site/region, where the protein interacts to execute its chaperone activity. For this purpose, the adenine nucleoside at either the 6th, or 11th or 20th position in the 5UAR hairpin was replaced with a 2-amino purine (2Ap) residue (Figure S3A). 2Ap is a fluorescent analogue of adenosine and site-selectively reports on the dynamics of interaction with proteins through fluorescence measurements [59]. In case of 5UAR, increase in the 2Ap fluorescence upon protein binding would indicate origin of structural or dynamic alteration in the RNA hairpin. The replaced residue at the 6th, 11th and 20th position represents the stem region near the base of the 5UAR loop, the region in the 5UAR loop and the 5UAR lower stem, respectively. We observed a gradual enhancement in 2-aminopurine fluorescence at the 20th position with increasing concentrations of either Denv2C or Denv2C^S34C^. This observation suggests that both proteins actively interact with the lower stem region of the 5UAR hairpin (Figure S3B and S3C). Such interactions result in the destabilization and unwinding of hydrogen-bonded base pairs within the RNA stem, consistent with fraying and melting phenomena leading to the annealing of nucleic acids [12]. Notably, the increase in 2AP fluorescence was ~ 2-fold lower in the presence of Denv2C^S34C^, highlighting the potential impact of the S34C mutation on Denv2C’s binding dynamics (Figure S3C).

We further explored the interaction between Denv2C and the 5UAR by conducting all-atom molecular dynamics (MD) simulations to identify the key determinants of this interaction. Since 5UAR exists in both partially open and fully open conformations [12], we investigated both the folded (5UAR_F_) and the linear (5UAR_L_) states of 5UAR hairpin in the presence and absence of Denv2C. We initially positioned the folded 5UAR (5UAR_F_) in proximity to the α4-helix of the Denv2C dimer and performed MD simulations of the complex (Figure S4A). Remarkably, within 10 ns, binding between 5UAR_F_ and Denv2C was observed across all replicate simulations. This binding induced significant structural alterations in 5UAR_F_, characterized by a notable reduction in intra-RNA H-bonding (Figure S4B-S4C). Additionally, the interaction caused pronounced structural fluctuations in Denv2C as well, when bound to 5UAR_F_ (Figure S4B-S4C).

Detailed trajectory analysis revealed that Lys and Arg residues within the α4-helices formed hydrogen bonds with 5UAR (Figure S4D). These residues also engaged in electrostatic interactions with the phosphate backbone atoms of 5UAR_F_, originating from both the α4-helices and intrinsically disordered regions (1–24 amino acids) of Denv2C (Figure S4D-F). Furthermore, Denv2C binding induced localized structural changes in 5UAR_F_, leading to a reduction in base pair H-bonds and loss of contacts in the stem region (Figure S5A). Notably, in two out of three Denv2C:5UAR_F_ simulation replicates, Denv2C binding triggered unzipping of the 5UAR stem region (Figure S5A-B), a phenomenon absent in all protein-free 5UAR_F_ simulation replicates. Comparative analysis of base-pair and base-step parameters between free and Denv2C-bound 5UAR_F_ further demonstrated that Denv2C specifically perturbs base pairs in the stem region (Figure S5C). Collectively, these findings show that the electrostatic interactions between Lys and Arg residues of Denv2C and the phosphate backbone of 5UAR play a critical role in destabilizing and unzipping the 5UAR stem structure, a pre-requisite for annealing of complementary nucleic acids.

Furthermore, similar MD simulations with the linear 5UAR (5UAR_L_) exhibited reduced structural fluctuations and a decreased radius of gyration when bound to Denv2C compared to its behavior in the absence of the protein during the simulation period (Figure S6). Additionally, time-averaged contact maps revealed a higher frequency of inter-RNA contacts in the absence of Denv2C (Figure S6D), indicating that Denv2C binding effectively resolves non-native inter-RNA interactions.

### Denv2C^S34C^ exhibits diminished ability for nucleic acid folding compared to Denv2C

To further investigate how Denv2C influences or resolves inter-RNA interactions, we employed single-molecule FRET (smFRET) assays. This approach allowed us to examine how Denv2C and its structural flexibility regulate nucleic acid folding dynamics at the single molecule level. During the vRNA rearrangement, RNAs navigate through folding and unfolding kinetics until they fold into their compact, thermodynamically stable and biologically active conformations. To examine these dynamics, we analyzed the single-molecule folding kinetics of a model 60-nucleotide stem-loop hairpin (Figure S2) [38] in both its ribose, RNA, (HPr) and deoxyribose, DNA, (HPd) form. These studies were conducted in the absence and presence of either Denv2C or Denv2C^S34C^ under physiological conditions. Similar to the 5UAR hairpin, the stem region of this surface-tethered ubiquitous nucleic acid structural motif (HPr and HPd) consists of 7–8 nucleotide base pairs (bp) (compare Fig. 1 and Figure S2) which exhibit dynamic folding and unfolding behavior [38].

**Fig. 3:**
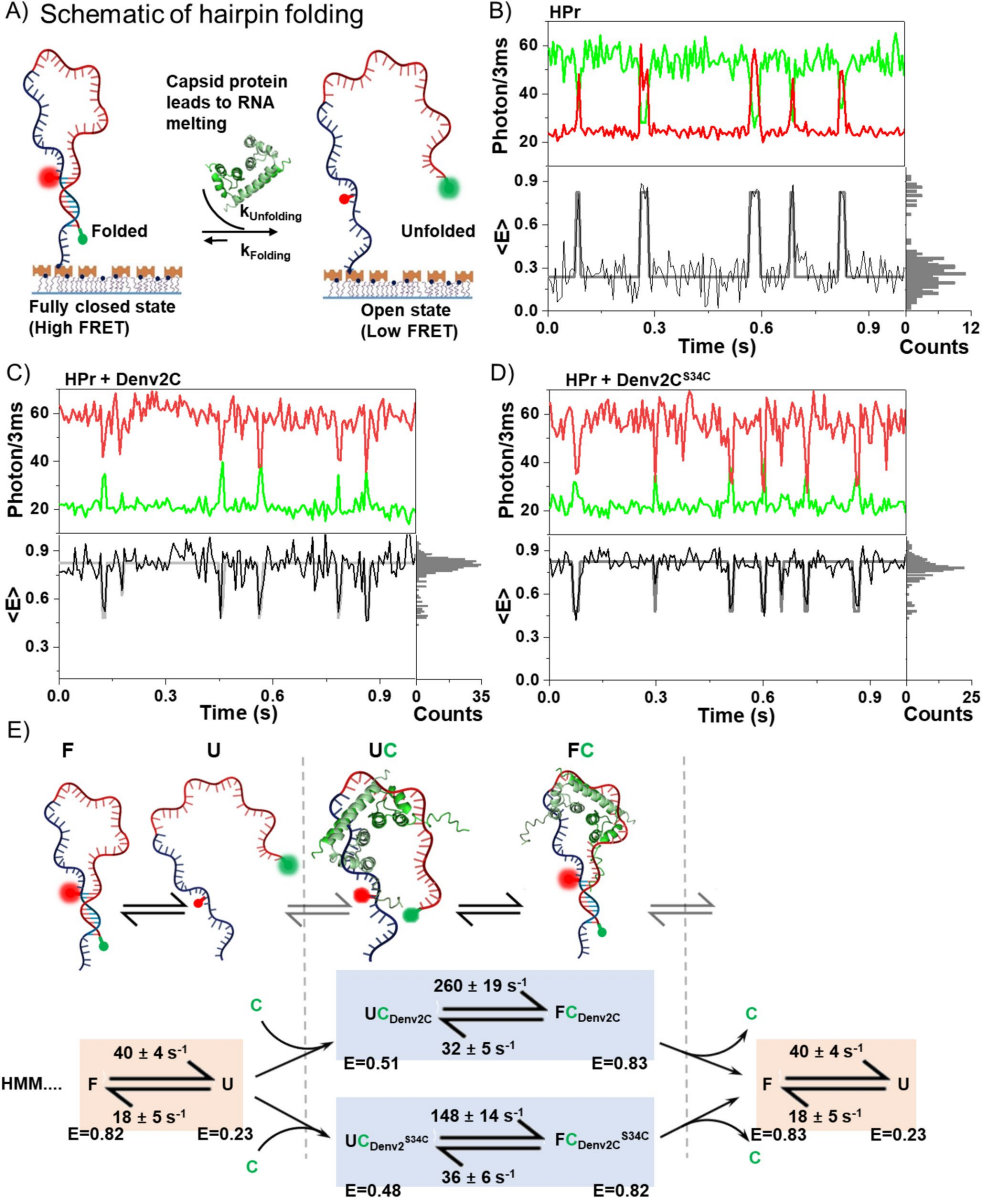
Chaperone-accelerated hairpin folding affected by structured domain. (**A**) Schematic representations of the folded and unfolded hairpins under smFRET experimental conditions. The biotinylated donor (green sphere)-acceptor (red sphere)-labelled hairpins were immobilized on PEGylated coverslips through a streptavidin–biotin interaction. The changes in the intensities of green and red spheres indicate the open (low FRET) and close (high FRET) between 5’ − 3’ ends of hairpin, either in the absence or presence of chaperone protein (shown by green ribbon cartoon). (**B**) Representative donor (green) and acceptor (red) fluorescence time traces depicting unassisted folding of the surface-immobilized 5′-3′ FRET-labelled RNA hairpin (HPr). The uncorrected transfer efficiency, E_FRET_, (black) and the most likely state trajectory (gray) based on the hidden markov model (HMM) are shown in the panel below. Analogous to (**B**) but depicting (**C**) Denv2C-assisted and (**D**) Denv2C^S34C^-assisted HPr folding with saturating concentrations of protein (200 nM) to ensure that HPr molecules are almost always chaperone-associated. (**E**) Kinetic 4-state model for Denv2C and its mutant-assisted folding. The folded, F, and unfolded, U, conformations (shown as cartoon) of the donor-acceptor labelled HPr freely interconvert in the absence of chaperone protein, **C**, (shown as green ribbon structure) with an equilibrium constant that favors U. When the chaperone protein is bound, the unfolded, UC (UC_Denv2C_ and UC_Denv2C_^S34C^), and folded, FC (FC_Denv2C_ and FC_Denv2C_^S34C^), conformations of the hairpin still interconvert, but with an equilibrium constant that favors F. However, that favorable equilibrium towards F conformation reduced by ~ 2-fold during Denv2C^S34C^-assisted folding as compared to Denv2C-assisted folding of HPr. All obtained values of transfer efficiency (< E>) and associated transition rates (k) are reported in Table 1.

We site-specifically labelled the hairpin molecules near the 3’ and 5’ ends with Cy3 and atto647N dyes, respectively, enabling the monitoring of stem-loop folding through changes in FRET efficiency. Fluorescence time traces of individual surface-immobilized 5’-3’ FRET-labelled hairpin molecules were recorded to capture their dynamic folding behavior (Fig. 3 and S7). In the absence of protein, both HPr (Fig. 3B) and HPd (Figure S7A & S7B) molecules transitioned freely between two conformations, characterized by low-FRET (< E_U_>) and high-FRET (< E_F_>) values of ~ 0.2 and ~ 0.8, respectively (Fig. 3 and S7). The interconversion rate (k_U◊F_) from the low-FRET unfolded (U) state to the high-FRET folded (F) state was ~ 2-fold slower than the reverse interconversion rate (k_F◊U_) with an equilibrium constant that favors the unfolded state (Fig. 3, S7 and Table 1). Interestingly, lowering monovalent ion concentrations (Na^+^: from 150 mM to 30 mM) or divalent ion concentrations (Mg^2+^: from 1 mM to 0.2 mM) did not alter either the interconversion rates or the equilibrium constant favoring the unfolded state of the nucleic acid hairpins (Figure S7A, S7B and Table 1). However, a reduction in mean transfer efficiency was observed, indicative of weakened electrostatic interaction between positively charged ions and the negatively charge nucleic acid phosphate backbone. These interactions are essential for stabilizing the compact and specific three-dimensional structures of nucleic acids [60].

**Table 1:** Transition rate constants for two kinetic regimes. Rate constants were obtained by fitting dwell time histograms of either folded conformation (~ 0.8 FRET value) or unfolded conformation (~ 0.2 FRET value) for hairpins in the absence of chaperone. Similarly, rate constants of hairpin in the presence of chaperone were obtained by fitting dwell time histograms of either folded conformation (~ 0.8 FRET value) or intermediate unfolded conformation (~ 0.5 FRET value). At least 60 molecules were observed for analysis

Oligonucleotide Conformation		Folded conformation (F)		Intermediate conformation (I)		Unfolded conformation (U)	
	RNA Chaperone	< E_F_>	k_F◊ U, I_ (s^− 1^)	< E_I_>	k_I◊ F_ (s^− 1^)	< E_U_>	k_U◊ F_ (s^− 1^)
	RNA hairpin (HPr)						
Buffer	-	0.82 (± 0.02)	40 (± 4)			0.23 (± 0.02)	18 (± 5)
	Denv2C ^a^	0.82 (± 0.03)	32 (± 5)	0.51 (± 0.03)	260 (± 19)		
	Denv2C^S34C a^	0.83 (± 0.04)	36 (± 6)	0.48 (± 0.02)	148 (± 14)		
	DNA hairpin (HPd)						
	-	0.82 (± 0.03)	42 (± 10)			0.22 (± 0.02)	25 (± 7)
	Denv2C ^a^	0.83 (± 0.02)	37 (± 12)	0.47 (± 0.03)	294 (± 54)		
	Denv2C^S34C a^	0.82 (± 0.03)	28 (± 4)	0.48 (± 0.03)	140 (± 24)		
	Denv2C ^b^	0.83 (± 0.02)	33 (± 11)	0.51 (± 0.02)	267 (± 46)		
	Denv2C^S34C b^	0.82 (± 0.02)	36 (± 4)	0.51 (± 0.04)	160 (± 16)		
Buffer^*^	-	0.82 (± 0.02)	36 (± 15)			0.22 (± 0.02)	22 (± 8)
	Denv2C ^a^	0.82 (± 0.02)	30 (± 8)	0.43 (± 0.02)	280 (± 105)		

^*^ Buffer containing 50 mM HEPES, 30 mM NaCl, 0.2 mM MgCl_2_, pH 7.5

^a Added protein at 200 nM concentration^

^b Added protein at 2 µM concentration^

At a concentration of 200 nM, both Denv2C and Denv2C^S34C^ induced significant differences in the fluorescence time traces compared to the absence of protein (Fig. 3C and D, S7C, S7D; Table 1). Both proteins preferentially stabilize the chaperone-bound folded conformation (FC) of the RNA as well as the DNA hairpin, reflected in the high-transfer efficiency value of ~ 0.8 (Fig. 3E and S7E). Additionally, there were frequent short excursions to a second conformation, characterized by an average dwell time of ~ 3 to 6 ms, leading to a minor population with a transfer efficiency of ~ 0.5 (< E_I_>) (Fig. 3C and D, S7C, S7D; Table 1). These findings indicate that the protein-bound unfolded hairpin (UC) is transiently populated at equilibrium, representing a more compacted form than the protein-free unfolded hairpin (U).

MD simulations further substantiated the structural compaction of the Denv2C bound HPr (Figure S8A-S8B), revealing that the presence of Denv2C significantly restricts the radius of gyration (Figure S8C, S8D), enhances inter-RNA interactions, and dampens RNA structural fluctuations (Figure S8E-S8G). These findings underscored the stabilizing role of Denv2C in modulating RNA conformations and dynamics. A comparison of the rate constants across the two distinct kinetic regimes revealed that both RNA (260 ± 19 s^-1^) and DNA (294 ± 54 s^-1^) hairpin folding rates are accelerated ~ 20-fold upon binding of Denv2C, with no significant change in the unfolding rate constants (Table 1). Upon the addition of 200 nM Den2C^S34C^, a notable decrease of ~ 2 to 3-fold in hairpin folding rates was observed– dropping from 260 ± 19 s^-1^ to 148 ± 14 s^-1^ for RNA hairpin (HPr) and from 294 ± 54 s^-1^ to 140 ± 24 s^-1^ for DNA hairpin (HPd), compared to folding rates in the presence of Denv2C (Table 1). Interestingly, no differences were observed in either protein-assisted short hairpin excursions (< E_I_>) or protein assisted-folding rates (k_I◊F_) when the protein concentration was increased from 200 nM to 2 µM (Table 1), indicating that the interaction between nucleic acid hairpins and Denv2C occurred under saturating conditions. Taken together, these results confirm that structural flexibility within the ordered Denv2C domain does not influence the protein assisted-conformations (both short (< E_I_>) and long (< E_F_> and < E_U_>) excursions of nucleic acids) but instead modulates the protein assisted-folding rates of the hairpin molecules.

### Reduced motion of the α1 − helices imparts rigidity to the ordered domain of Denv2C^S34C^

**Fig. 4:**
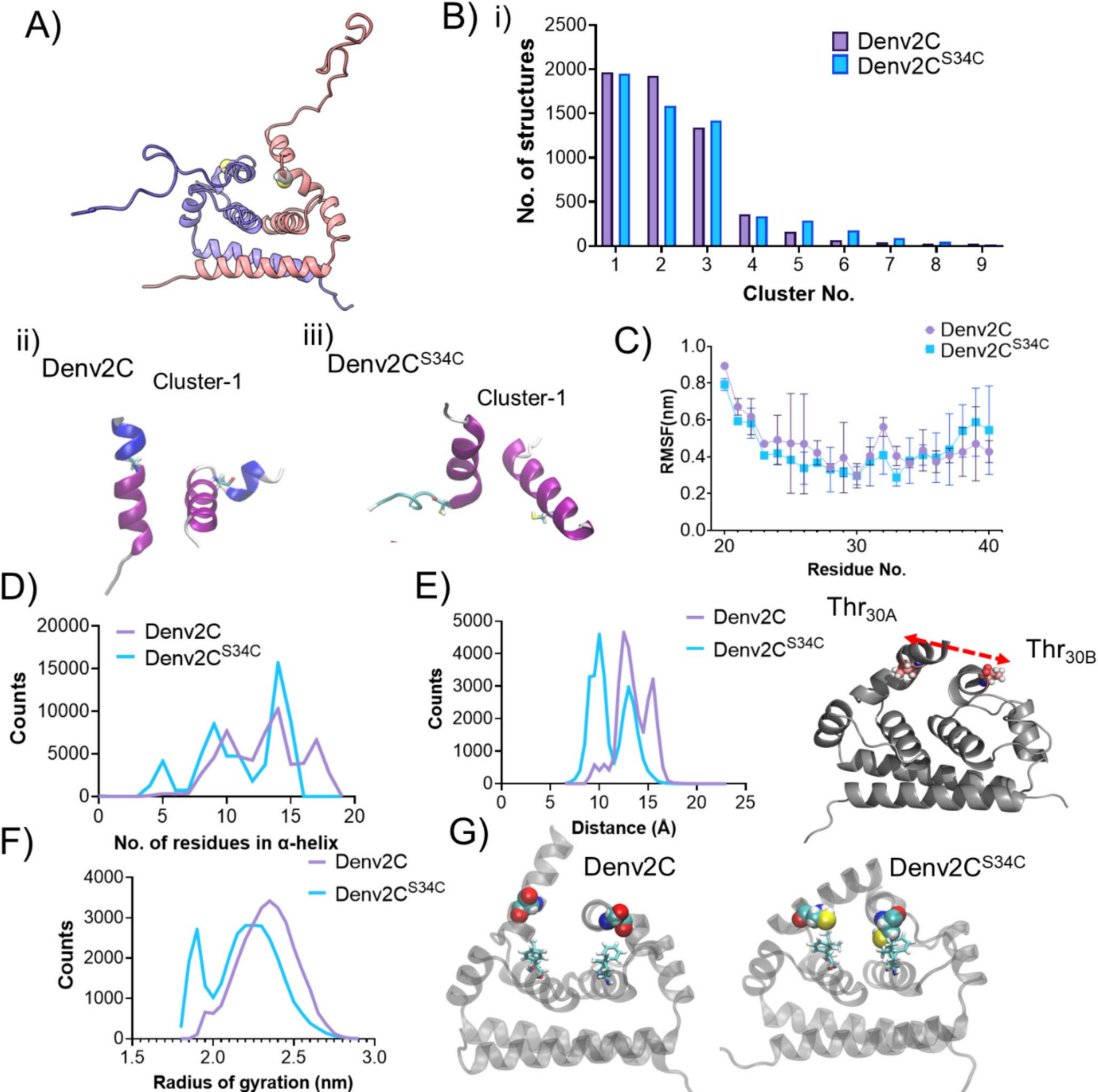
MD simulations reveal differences in dynamics of α1-helices and compactness of dimeric Denv2C and Denv2C^S34C^. **A**) Cartoon representation of C protein dimer highlighting residue 34 in sphere representation. **B**) Bar plots showing the number of structures in each cluster from Denv2C and Denv2C^S34C^ along with representative structures of folded region from most populated clusters of each system. Cluster analysis was performed on combined trajectories from three replicate 1000 ns simulations each for Denv2C and Denv2C^S34C^. C) Per-residue root mean square fluctuations (RMSFs) of region spanning residues 20–40, based on combined trajectories from three replicate 1000 ns simulations each for Denv2C and Denv2C^S34C^. **D**) Plot showing the distribution of number of amino acids existing in α-helical state in peptide region spanning residues 20–40 from each monomer of Denv2C and Denv2C^S34C^, calculated over the cumulative simulation time of 3000 ns. **E**) Plot showing the change in distance between T30 from each monomer of dimeric Denv2C and Denv2C^S34C^ over the cumulative simulation time of 3000 ns. **F**) Plot showing the distribution of radius of gyration of full length Denv2C and Denv2C^S34C^ over the cumulative simulation time of 3000 ns. **G**) Snapshots from Denv2C and Denv2C^S34C^ MD simulations trajectories showing residues 34 represented as spheres and proximal amino acids Leu_46_ and Phe_50_

Finally, to investigate the structural impact of S34C mutation in the ordered region of Denv2C, we performed all-atom MD simulations of dimeric Denv2C and Denv2C^S34C^ (Fig. 4A). An equilibrated full-length structure of Denv2C, previously reported [48], served as the basis for generating the Denv2C^S34C^ mutant. Simulations were performed for 1 µs for both proteins, with triplicate runs to enhance conformational sampling. The root mean square deviation (RMSD) of backbone atoms relative to their starting structures and the per-residue root mean square fluctuation (RMSF) profiles revealed high intrinsic flexibility in the disordered N-terminal region (1–24 amino acids) compared to the rigid, folded core region (24–100 amino acids) for both Denv2C and Denv2C^S34C^ (Figure S9A-B). While the helical core remained structurally stable, the α1-helices exhibited increased dynamics (Fig. 4).

To further investigate the conformational dynamics of the α1-helices, we performed cluster analysis on 3 µs of combined triplicate trajectories generated for each system. Significant differences were observed in the conformations of α1-helices between the three dominant clusters from Denv2C and Denv2C^S34C^ MD simulation trajectories (Fig. 4B and S10A). For the helical core (20–100 amino acids), the most populated cluster for Denv2C^S34C^ contained more than 3000 structures out of 6000 total structures, whereas Denv2C exhibited four highly populated clusters. This suggests that wild-type Denv2C may exhibit greater dynamics than Denv2C^S34C^, sampling multiple conformational states (Figure S10B-S10C). This conclusion was supported by an average reduction in flexibility of dominant motions around the α1-helices region in Denv2C^S34C^ (Figure S10D-E, 5 C). In addition, the region spanning the α1-helix contained a greater number of residues with helical structure in Denv2C^S34C^ (Fig. 4D) on average.

To further understand how the differences in α1-helical dynamics influenced the behavior of the dimeric protein as a whole, we examined their impact on overall conformational flexibility. Both Denv2C and Denv2C^S34C^ exhibited the characteristic “opening” and “closing” motions of the α1-helix within the ordered domain, as previously described [48], which regulate access to the central hydrophobic patch. To quantify the frequency of these transitions, we measured the distance between Thr_30_ residue from each monomer, as a proxy for the extent of structural flexibility. Remarkably, the α1-helices in Denv2C^S34C^ were capable of achieving significantly closer distances in the closed state compared to Denv2C (Fig. 4E and S10B). In addition, the α1-helices of Denv2C^S34C^ remained in a closed state for longer durations than those of native Denv2C throughout the simulation time (Fig. 4E). This behavior appeared to stem from the less polar nature of Cys_34_ compared to Ser_34_, allowing Cys_34_ to form stronger interactions with multiple hydrophobic residues in the core of the capsid protein dimer, including Leu_46_, Phe_50_ (Fig. 4G). Consequently, Denv2C^S34C^ adopted a more compact structure than native Denv2C, as shown by the radius of gyration distribution (Fig. 4F). This increased rigidity of the ordered region in Denv2C^S34C^ likely underpins its diminished chaperone activity compared to native Denv2C. This underscores the pivotal role of α1-helical flexibility in enabling the protein’s function as an RNA chaperone.

We assessed the intrinsic disorder profiles of the dengue virus capsid proteins, encompassing both ordered and disordered regions which determine a protein’s structural integrity and functional flexibility. This analysis was performed using the Critical Assessment of Intrinsic protein Disorder (CAID) prediction portal (https://caid.idpcentral.org/), which integrates multiple disorder prediction algorithms to assign a disorder probability score to each residue [61]. We evaluated the disorder propensities of native Denv2C (Figure S11A) and Denv2C^S34C^ (Figure S11B). The majority of prediction algorithms showed that the N-terminal and early middle regions (positions 1–24) exhibit high disorder scores (> 0.5), suggesting these regions are intrinsically disordered and align with their roles in membrane binding and cellular entry as well as RNA encapsidation [62] (Figure S11A). Notably, the region spanning positions 25–45 displayed intermediate disorder scores (~ 0.3–0.5), indicating it may feature mixed-order characteristics [63] (Figure S11A) - a signature often associated with conditionally disordered regions that may undergo conformational transitions. This region likely undergoes a disorder-to-order transition, essential during viral assembly, capsid maturation, and RNA binding [64]. To further probe this structural transition, we investigated the differences between the disorder profiles of native Denv2C and Denv2C^S34C^ (Figure S11C). The positive difference in residue-wise disorder score (i.e., native Denv2C minus Denv2C^S34C^) within residues ~ 25–40 confirms that the S34C mutation promotes a more compact and ordered conformation in this (Figure S11C). Capsid proteins from other dengue virus serotypes (Denv1C, Denv3C, and Denv4C) exhibited similar disorder profiles (Figure S11D) and displayed positive differences in residue-wise disorder scores when compared to Denv2C (Figure S11E). These findings suggest a potentially conserved role for the residue 34 in modulating capsid protein structural dynamics across serotypes, highlighting its relevance beyond serotype-specific contexts. Finally, the C-terminal region (residues 60–100) exhibited consistently low disorder scores (< 0.3), indicating a predominantly ordered domain that likely contributes to stable secondary structure and is essential for maintaining overall protein integrity [62, 64].

## Summary

Melting, annealing and strand displacement of conserved coding region elements like 5UAR enables dengue viral RNA to adopt diverse conformations essential for RNA synthesis. These RNA molecules are prone to forming stable and persistent alternative secondary structures, which must overcome significant thermodynamic barriers to achieve correct folding and fulfill their biological roles [65–68]. RNA chaperones, such as Denv2C, are crucial in resolving RNA misfolding by facilitating the escape from unfavorable kinetic traps [12, 22–37, 41]. These proteins can interact with both RNA and DNA polynucleotides due to their shared nucleic acid-binding properties, structural adaptability, and a function to modulate nucleic acid structures [15, 17, 57, 58]. This versatility is facilitated by their flexible, nonspecific nucleic acid-binding domains, enabling dynamic engagement with diverse nucleic acid substrates [15, 17, 57, 58]. To investigate Denv2C’s role, we analyzed the annealing and strand displacement kinetics of 5UAR with its complementary c5UAR. Harnessing its chaperone properties, Denv2C accelerates 5UAR/c5UAR annealing [12] while inducing strand displacement of nucleic acids [41].

**Fig. 5:**
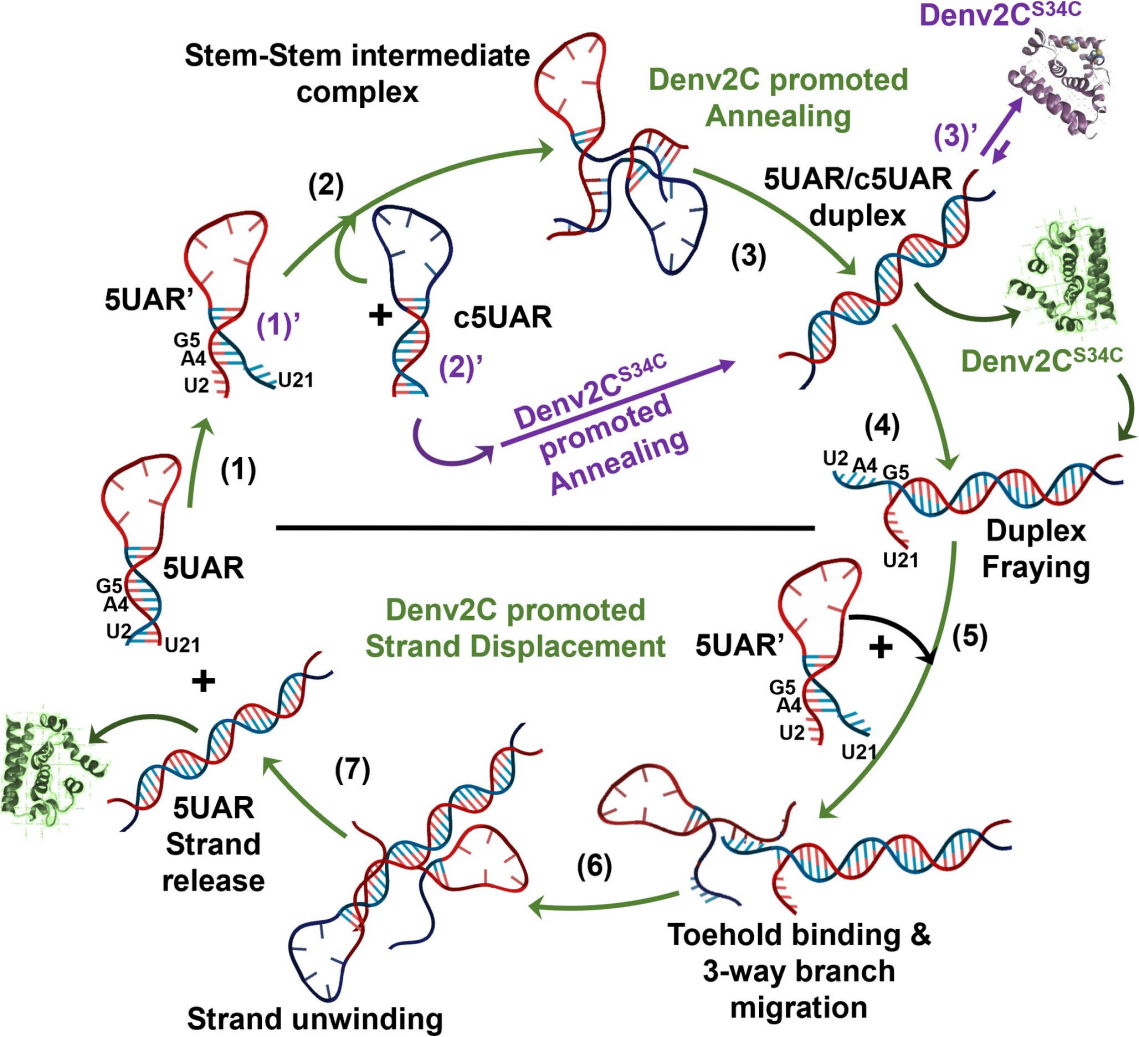
Proposed reaction mechanisms for the Denv2C-promoted 5UAR/cUAR (steps 1, 1’, 2 and 3), Denv2C^S34C^ (steps 1, 1’, 2’ and 3’) annealing and Denv2C-promoted strand displacement (steps 4, 5, 6 and 7). The reaction mechanism starts with the destabilization and melting of the 5UAR stem due to the transient binding of either Denv2C (green cartoon) (step 1) or Denv2C^S34C^ (violet cartoon) (step 1’). This leads to an equilibrium between at least two different species of the 5UAR hairpin (5UAR and 5UAR’) that originates due to opening of the 5UAR hairpin through the melting A_1_-U_21_, U_2_-A_20_, A_4_-U_18_ and G_5_-C_17_ base pairs. This melting of base pairs accelerates the annealing reaction in the presence of the complementary c5UAR hairpin (step 2, 3 and 3’). The 5UAR/c5UAR annealed duplex is formed via a stem-stem intermediate (step 3) in the presence of Denv2C while no such intermediate is observed in the presence of Denv2C^S34C^. Denv2C accelerates the 5UAR/c5UAR annealing reaction by ~ 80-folds when compared to Denv2C^S34C^ accelerated 5UAR/c5UAR annealing (steps 3’), due to its higher structural flexibility and lower transient binding time to nucleic acids. In the second half, Denv2C initiates and catalysis the strand displacement reaction in the presence of at least 10-fold molar excess of the displacing 5UAR by destabilizing the 5UAR/c5UAR duplex through its transient interaction with U_2_, A_4_, G_5_ and U_21_ nucleotides at either ends (step 4 and step 5) of duplex. This leads to melting of A_1_-U_21_, U_2_-A_20_, A_4_-U_18_ and G_5_-C_17_ base pairs in the 5UAR/c5UAR duplex and toehold binding of Denv2C-destabilized 5UAR (5UAR’) (step 5). Toehold binding leads to the nucleation of a transient intermediate triplex where the invader 5UAR strand (5UAR’) unwinds (step 6) and releases (step 7) the incumbent annealed 5UAR stand from the 5UAR/c5UAR duplex

Our results indicate that Denv2C-promoted annealing and strand displacement propagate through an active conformation of 5UAR, termed 5UAR’, in which the stem region is frayed due to interactions with nucleotides in the stem (Fig. 5). This fraying involves the melting of 4–5 base pairs (A_1_-U_21_, U_2_-A_20_, A_4_-U_18_ and G_5_-C_17_) in the 5UAR stem, forming a transient intermediate complex stabilized by up to 10 intermolecular base pairs before transitioning to a 5UAR/c5UAR duplex (Fig. 5) [12]. During annealing, Denv2C facilitates a microenvironment via RNA–protein and protein–protein interactions [57], acting as a “matchmaker” by reducing electrostatic repulsion and extending the lifetime of RNA–RNA complexes, thereby enhancing annealing efficiency [69].

In contrast, the strand displacement process is fundamentally distinct, involving an invading RNA strand binding to an annealed complex and displacing the incumbent strand. Denv2C catalyzes strand displacement by destabilizing the edges of annealed duplexes [41], likely by melting 4–5 base pairs in the 5UAR/c5UAR duplex. This mechanism involves the formation of a three-way branch migration intermediate triplex, arising through toe-hold binding of an invading 5UAR’ sequence, followed by unravelling and release of the donor-labelled 5UAR (Fig. 5). According to the “entropy exchange model,” cyclic RNA binding and release, coupled with reciprocal entropy transfer between RNA and protein, drives RNA folding into its most stable secondary structure [70]. Therefore, during strand displacement, Denv2C probably performs a “conformational search” of the target RNA [15] via successive cycles of transient binding.

Our results showed that Denv2C switches the conformational equilibrium of nucleic acid hairpins from an adverse unfolded state to the favorable folded and compacted state, resulting in their disorder-to-order transition. Interestingly, RNA chaperones like Denv2C [22–37] exhibit structural plasticity, with either predominantly folded domains (e.g., NCp7) or regions combining stably ordered areas (for RNA recognition) with intrinsically disordered regions that act as flexible macromolecular counterions to screen repulsive electrostatic interactions [17, 38, 58]. While IDRs are essential for RNA annealing activity, the role of ordered regions remains debated.

To address this, we monitored the 5UAR disorder-to-order transition (annealing, folding, unfolding, and strand displacement) in the presence of Denv2C^S34C^, focusing on the role of the ordered region. We observed that loss of flexibility in the ordered region significantly impaired Denv2C’s RNA chaperone activity, resulting in a ~ 80-fold slower 5UAR/c5UAR annealing rate (Supplementary Table M1). This reduced activity stemmed from slower RNA folding rates and a diminished ability for successive cycles of transient RNA binding (Figure S12). Thus, flexibility in the ordered region, in conjunction with intrinsically disordered regions, is crucial for Denv2C to perform complex strand displacement reactions. Interestingly, when Denv2C loses flexibility in its ordered region, it behaves more like an annealer, swapping nucleic acid melting ability for aggregation propensity [12, 32, 40, 41, 71–73]. This is consistent with Denv2C acting as an unstructured polyelectrolyte that screens electrostatic interactions equivalently to molar salt concentrations [38]. This confirms that structural changes in the ordered region govern Denv2C’s RNA chaperone function.

We propose an ensemble mechanism for multimeric RNA chaperones, wherein the disordered region acts as a macromolecular counterion [38] and the flexible ordered regions provide specificity for RNA conformational transitions. The ability of Denv2C to modulate (+) RNA circularization by controlling annealing and strand displacement mechanisms of conserved RNA elements expand our understanding of the molecular strategies the virus employs for genome rearrangement, replication, and evasion of antiviral responses.

## Electronic supplementary material

Below is the link to the electronic supplementary material.

Supplementary Material 1

## Data Availability

The authors confirm that the data supporting the findings of this study are available within the article and its supplementary material. Raw data that support the findings of this study areavailable from the corresponding author upon request.
