# Supplementary material for: Structural flexibility in the ordered domain of the dengue virus strain 2 capsid protein is critical for chaperoning viral RNA replication
Source: Cell Mol Life Sci. 2025 Apr 28;82(1):184. doi: 10.1007/s00018-025-05712-x (PMC12037954; doi:10.1007/s00018-025-05712-x)
Supplement: Supplementary file 1 — Supplementary Material 1 [file 18_2025_5712_MOESM1_ESM.docx]

**Structural flexibility in the ordered domain of the dengue virus strain 2 capsid protein is critical for chaperoning viral RNA replication**

Kamal K. Sharma^1,2*^, Palur Venkata Raghuvamsi^3^, Daniel Y. K. Aik^1,4^, Jan K Marzinek^3^, Peter J. Bond^3^ , Thorsten Wohland^1,2,4*^,

^1^Centre for Bioimaging Sciences, National University of Singapore, 14 Science Drive 4, Singapore 117557, Singapore.

^2^Department of Biological Sciences, National University of Singapore, 14 Science Drive 4, Singapore 117543, Singapore.

^3^Bioinformatics Institute (BII), Agency for Science, Technology and Research (A*STAR), 30 Biopolis Street, #07-01 Matrix, Singapore 138671, Republic of Singapore.

^4^Department of Chemistry, National University of Singapore, 3 Science Drive 3, Singapore 117543, Singapore.

*To whom correspondence should be addressed: Kamal K. Sharma, Tel: +65 82906620; Email: [dbskks@nus.edu.sg](mailto:dbskks@nus.edu.sg) or Thorsten Wohland. Tel: +65 65161248; Email: [twohland@nus.edu.sg](mailto:twohland@nus.edu.sg).


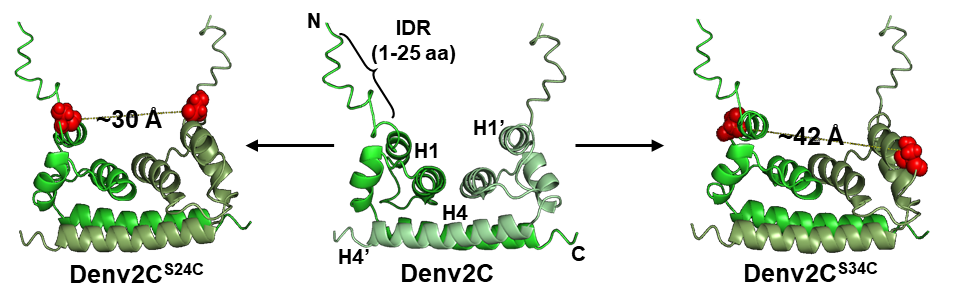


**Figure S1:** Schematic representation of Denv2C and its mutants. Denv2C structure was prepared by modifying 1R6R structure from the protein data bank. Intrinsically disordered region (1-25 amino acids), IDR, were added to each monomeric unit while while rest of the residues (22-100 amino acids) constitute the ordered region. The α1 helix (H1) and α4 helix (H4) are labelled for both subunits of the protein, with H1 and H4 from one subunit and H1ʹ and H4ʹ from the other subunit of Denv2C. Denv2C^S24C^ and Denv2C^S34C^ mutants were created by replacing serine residues with cysteine residue at position 24 and 34 (Shown as red spheres), respectively.


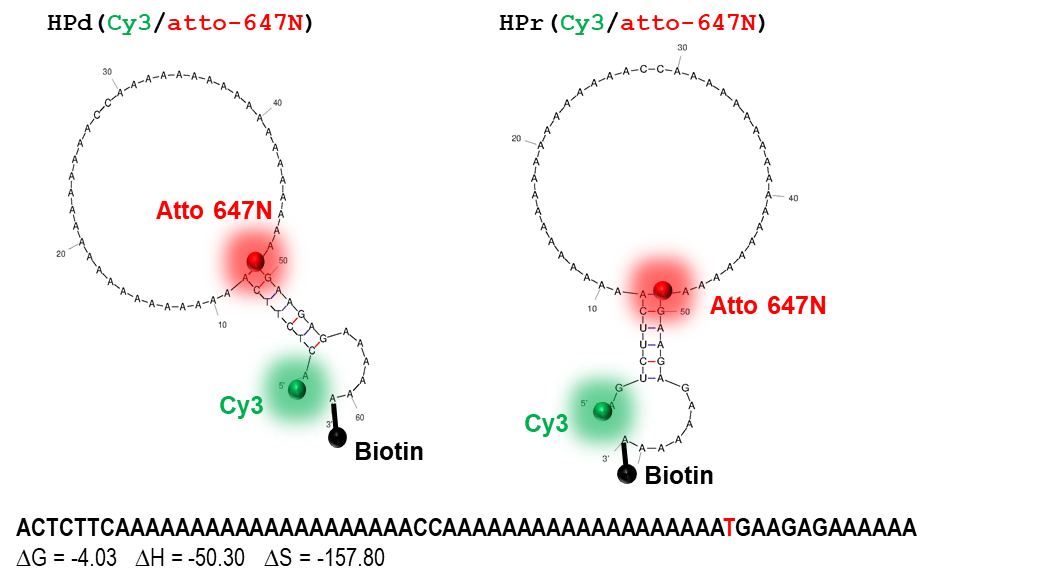


**Figure S2:** Schematic representation of ubiquitous nucleic acid structural motif - a stem-loop hairpin (HP). Both RNA and DNA form of HPs sequences are inspired from the work of Erik D. Holmstrom et al. [1]. Their secondary structures were predicted using the mfold webtool (<http://unafold.rna.albany.edu/>). The doubly-labelled RNA based hairpin (HPr) as well as DNA based hairpin (HPd) were synthesized with Atto647N (red sphere), cyanine3 (green sphere) and biotin (black sphere) at the 5’end, at the 48^th^ position towards 3’ end and at the 3’ end, respectively.


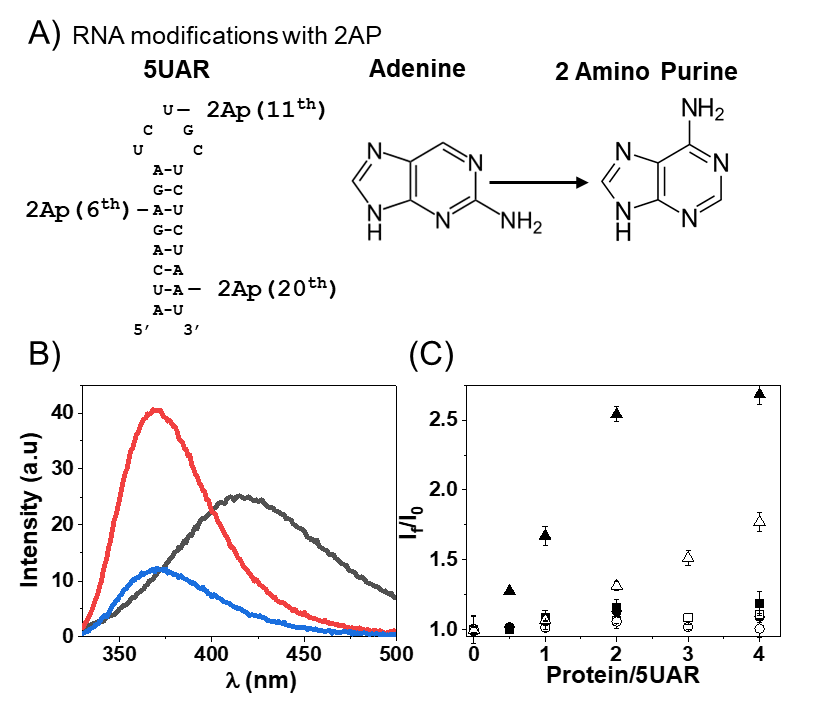


**Figure S3:** Binding site Denv2C and Denv2C^S34C^ on 5UAR sequence. To monitor binding Denv2C binding site on RNA based 5UAR hairpin, (A) adenine nucleosides at 6^th^, 11^th^ and 20^th^ positions were replaced with 2-amino purine (2Ap) residues (shown as chemical structure). The replaced residues represents stem region near base of 5UAR loop, 5UAR loop and 5UAR lower stem regions, respectively. 2Ap is a fluorescent analogue of adenosine that minimally disturbs the nucleic acid structures and can site-selectively report on the dynamics of interaction with proteins through fluorescence measurements [2, 3]. (B) Emission spectra of 2Ap labelled 5UAR at positions 6^th^ (black), 11^th^ (red) and 20^th^ (blue). Different emission maxima of 2Ap at 3 positions of 5UAR suggests distinct environment experienced by the fluorophore, nucleotide in loops being the most solvent exposed. (C) Graph showing gradual increase in the 2Ap florescence at positions 6^th^ (squares), 11^th^ (circles) and 20^th^ (triangles) with increasing concentrations of either Denv2C (filled symbols) or Denv2C^S34C^ (open symbols). Both proteins induced an increase in 2Ap fluorescence at 20^th^ nucleotide position (filled and open triangles) suggesting that interaction taking place in the stem region of 5UAR hairpin. However, the effect of Denv2C^S34C^ is ~2-fold lower as compared to Denv2C again indicating the role of structured domain of Denv2C in this interaction.

**Table S1: MD simulation system setup**

| **System** | | **Water molecules** | **Ion concentration** | | **Box size** | **Production run (ns)** |
| --- | --- | --- | --- | --- | --- | --- |
| Denv2C/HPr | Orientation 1(O1) | 158333 | | 150mM NaCl,  1mM MgCl^2^ | 17.2 × 17.2 × 17.2 | 250 |
|  | Orientation 2(O2) | 169238 | |  | 17.4 × 17.4 × 17.4 | 250 |
|  | Orientation 3(O3) | 169238 | |  | 17.4 × 17.4 × 17.4 | 250 |
|  |  |  | |  |  |  |
| Denv2C/5UAR (Linear) | Orientation 1(O1) | 55390 | |  | 12.0 × 12.0 × 12.0 | 250 |
|  | Orientation 2(O2) | 59410 | |  | 12.3 × 12.3 × 12.3 | 250 |
|  | Orientation 3(O3) | 34505 | |  | 10.5 × 10.5 × 10.5 | 250 |
| Denv2C | | 43602 | 150mM NaCl,  1mM MgCl^2^ | | 11.1 × 11.1 × 11.1 | 3×1000 |
| Denv2C^S34C^ | | 41471 |  |  | 11.1 × 11.1 × 11.1 | 3×1000 |
| HPr (Linear) | | 168004 |  |  | 17.5 × 17.5 × 17.5 | 3×250 |
| 5UAR_L_ (Linear) | | 33055 |  |  | 7.2 × 7.2× 7.2 | 3×250 |
| 5UAR_F_ (Folded) | | 11886 |  |  | 7.2 × 7.2× 7.2 | 3×1000 |
| Denv2C/5UAR_F_ | | 57867 |  |  | 12.2 × 12.2 × 12.2 | 3×1000 |

**M1: 5UAR/c5UAR annealing mechanism in the presence of Denv2C and Denv2C^S34C^**

We monitored 5UAR/c5UAR annealing kinetics in the presence of either Denv2C or Denv2C^S34C^ (Figure M1). Observing the Cy3 fluorescence of donor-labelled 5UAR in the presence of Atto647N-labelled c5UAR during real time annealing kinetics shows a continuous decrease in FRET before reaching a plateau with the formation of the duplex that contains both Cy3 and Atto647N in proximity (Figure M1A). We fitted the real-time fluorescence intensity traces of the Denv2C-promoted 5UAR/c5UAR reaction kinetics (Figure M1A) using bi-exponential equation [1]

, [1]

$$I\left( t \right)=I_{f}-\left( I_{f}{-I}_{0} \right)\left( ae^{\left( -k_{obs1}\left( t-t0 \right) \right)}+\left( 1-a \right)e^{\left( -k_{obs2}\left( t-t0 \right) \right)} \right)$$

where *I(t)* is the actual fluorescence intensity at 560 nm, upon excitation at 532 nm, *k*_obs1_ and *k*_obs2_ are the pseudo first order fast and slow reaction rates, *a* is the relative amplitude of the fast component and *t*_0_ is the start time of the reaction. *I_0_* and *I_f_* are the fluorescence intensities of donor‑labelled 5UAR in the free state and in the final extended duplex (ED) in the presence of acceptor-labelled c5UAR, respectively.

On the other hand, fluorescence traces of the Denv2C^S34C^-promoted 5UAR/c5UAR reaction kinetics (Figure MS1D) were fitted to a mono-exponential equation [2]

[2]

$$I\left( t \right)=I_{f}-\left( I_{f}{-I}_{0} \right)\left( e^{\left( -k_{\mathrm{obs}}\left( t-t0 \right) \right)} \right)$$

Here k_obs_ is the second order reaction rate.

The fast and slow reaction rates of Denv2C-promoted 5UAR/c5UAR annealing, *k*_obs1_ and *k*_obs2,_ were plotted against the concentration of acceptor labelled-c5UAR reactant (Figure MS1B and MS1C). The fast component, *k*_obs1_, linearly varies with increasing c5UAR concentrations ([c5UAR-atto647N]) (Figure M1B), while the slow component, *k*_obs2_, shows a hyperbolic dependence (Figure M1C). Conversely, the Denv2C^S34C^-promoted 5UAR/c5UAR annealing reaction rates, *k_obs_*, shows a liner dependence with increasing c5UAR concentrations ([c5UAR-atto647N) (Figure M1E).

The linear relationship between *k*_obs1_ or *k_obs_* and [c5UAR-atto647N] follows equation [3] [4], where *k_ass_* is a second order association rate constant and *k_diss_* represents the first order dissociation rate constant.

[3]

$$k_{obs1} or k_{\mathrm{obs}}=k_{\mathrm{ass}}\left[ c5UAR-atto647N \right]+k_{\mathrm{diss}}$$

The hyperbolic dependence of *k*_obs2_ can be described by equation [4]

[4]

$$k_{obs2}=\frac{k_{f}K_{M}\left[ c5UAR-atto647N \right]}{1+K_{M}\left[ c5UAR-atto647N \right]}+k_{b}$$

where *K*_M_ is the equilibrium constant governing intermediate complex (IC) formation, *k*_f_ and *k*_b_ are forward and backward interconversion kinetic rate constants.

Fitting the linear (for both Denv2C- and Denv2C^S34C^-promoted) and hyperbolic (for Denv2C-promoted) plots against increasing [c5UAR-atto647N] with equations [3] and [4], respectively, resulted in the kinetic parameters shown in Table M1.

Based on the acquired kinetic parameters, we propose a reaction mechanism to follow a single kinetic pathway starting from a 5UAR species for Denv2C-promoted 5UAR/c5UAR annealing:


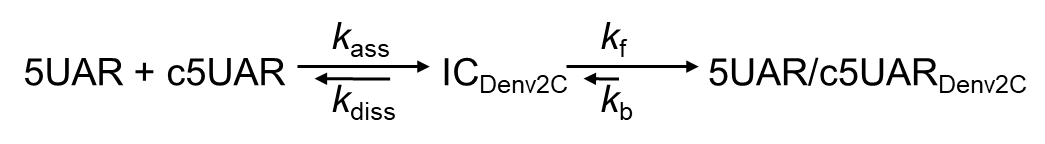
 Scheme 1

where a fast pre-equilibrium intermediate complex, IC_Denv2C_, precedes the formation of the final stable extended duplex, 5UAR/c5UAR_Denv2C_, through a monomolecular reaction. The formation of IC_Denv2C_ is governed by the second order association constant, *k*_ass_, and the first order dissociation constant, *k*_diss_, whereas the formation of 5UAR/c5UAR _Denv2C_ is governed by the forward and backward interconversion kinetic rate constants, *k*_f_ and *k*_b_ respectively. The hyperbolic dependence of *k*_obs2_ with increasing [c5UAR-atto647N] showed the IC_Denv2C_ accumulation because of its slow and rate limiting interconversion to 5UAR/c5UAR _Denv2C_.

Similarly, we propose the reaction mechanism of Denv2C^S34C^-promoted 5UAR/c5UAR annealing to follow a single kinetic pathway:


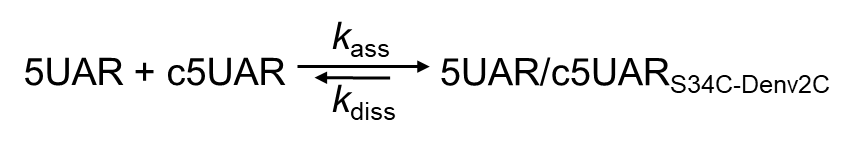
 Scheme 2

where the final stable extended duplex, 5UAR/c5UAR_Denv2C_^S34C^, is formed through a bimolecular reaction [5-7] and is governed by the second order association constant, *k*_ass_, and the first order dissociation constant, *k*_diss_, respectively.


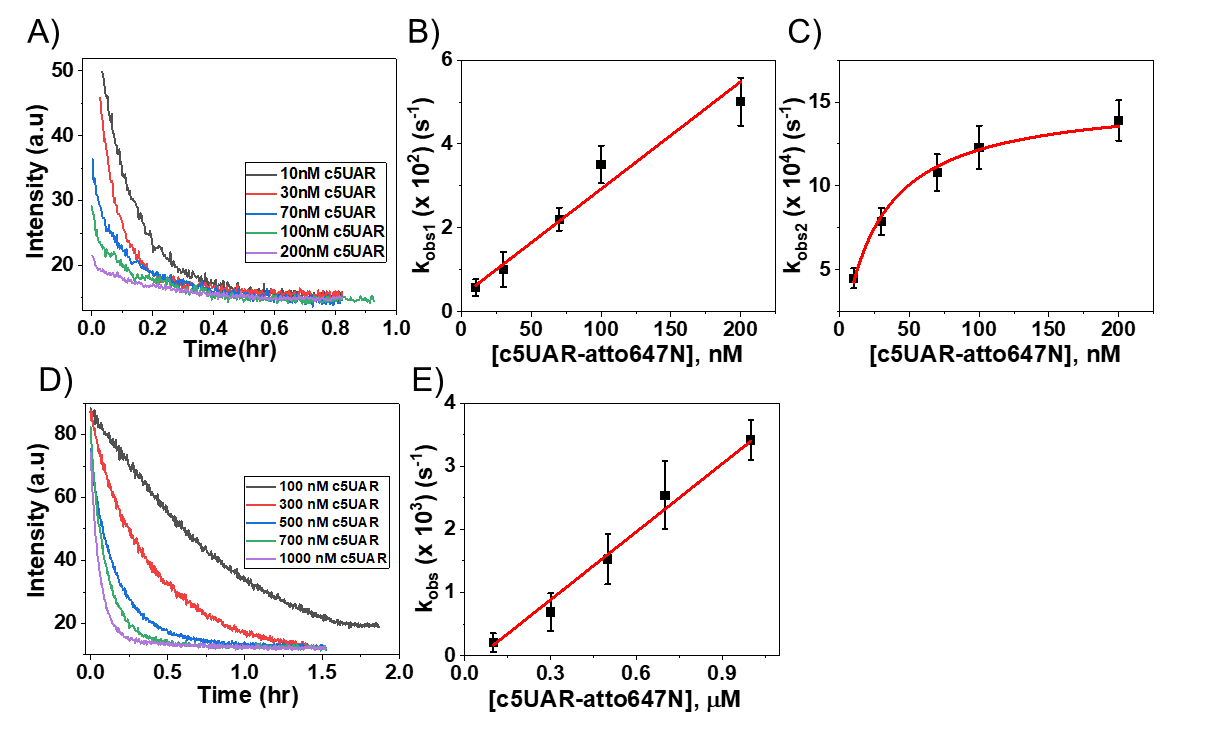


**Figure M1:** Real time progress curves and kinetic parameters of 5UAR/c5UAR annealing in presence of Denv2C (A, B and C) and Denv2C^S34C^ (D and E). Progress curves of 10 nM Cy3-labelled 5UAR annealing with increasing concentrations of Atto647N-labelled c5UAR (showed in the legend) in the presence of either (A) Denv2C or (B) Denv2C^S34C^ at a protein:ORN ratio 4:1. Excitation and emission wavelengths were 532 nm and 560 nm, respectively. Denv2C-assisted and Denv2C^S34C^-assisted 5UAR/c5UAR annealing traces were fitted using equation (1) and equation (2). Obtained (B) fast, k_obs1_, and (C) slow, k_obs2_, kinetic rates in the presence of Denv2C were plotted against increasing concentrations of the complementary Atto647N-labelled c5UAR and fitted using equation (3) and (4), respectively. Similarly, kinetic rates observed in the presence of (E) Denv2C^S34C^ were plotted and fitted using equation (3). All the obtained values are provided in Table M1.

In addition to changes in their kinetic pathways, the Denv2C-promoted 5UAR/c5UAR annealing reaction was ~80-fold faster as compared to the Denv2C^S34C^-promoted 5UAR/c5UAR annealing reaction (by comparing corresponding k_ass_ values in Table M1). These results indicate that the S34C mutation in Denv2C is probably able to alter structural properties of the Denv2C ordered region that may alter the chaperoning ability of the protein, also supported by our simulation results in later section. Interestingly at higher Denv2C:ORN ratio, a ~30-fold increase in the value of k_ass_ indicates the molecular crowding effect leading to higher charge screening of nucleic acids [1].

**Table M1:** **Kinetic parameters of 5UAR/c5UAR annealing in the absence and presence of either Denv2C or Denv2C^S34C^.** Kinetic rate constants, k_ass_, k_diss_, Ka and k_f_, were calculated from the dependence of the k_obs_ values on the concentration of the Atto647N-labelled c5UAR, using equations [3] and [4] as indicated in Figure M1. The K_a_ values were found to differ by a factor of <1.8 from the k_ass_/k_diss_ values, which further supports the proposed reaction Scheme 1.

| **Cy3-**  **labelled**  **ORN** | **Atto647N-**  **labelled**  **complementary**  **ORN** | **Protein:ORN**  **ratio** | ***k*_ass_ (M^−1^s^−1^)**  **×10^−3^** | ***k*_diss_ (s^−1^)**  **×10^4^** | **K_a_ (M^−1^)**  **×10^−5^** | ***k*_f_ (s^−1^)**  **× 10^4^** |
| --- | --- | --- | --- | --- | --- | --- |
|  |  |  |  | **Denv2C** |  |  |
| 5UAR | c5UAR | 0 ^a^ | 0.05(±0.003) | 3.6(±0.7) | 1.11(±0.19) | 2.02(±0.17) |
|  |  | 2 ^a^ | 8.4(±1.3) | 35(±15) | 16.8(±5.1) | 9.6(±1.1) |
|  |  | 4 | 255(±25) | 38(±17) | 385(±40) | 15.3(±0.5) |
|  |  | **Denv2C^S34C^** | | | | |
|  |  | 4 | 3.5(±0.2) | 1.9(±0.7) |  | |

^a^ kinetic parameters for comparisons are stated from earlier work done from our group [8]

**M2: 5UAR/c5UAR stand displacement mechanism in the presence of Denv2C and Denv2C^S34C^**

We monitored strand displacement kinetics of Cy3-labelled 5UAR from preformed Cy3/atto647N-labelled 5UAR/c5UAR duplexes. The addition of non-labelled 5UAR to the preformed duplexes in at least 10-fold molar excess (pseudo-first order conditions) should displace Cy3-labelled 5UAR leading to an increase in the Cy3 fluorescence (Figure 1C). Addition of non-labelled 5UAR (5UAR-nl), even at 10-fold excess, leads to a rapid increase in Cy3 fluorescence in the presence of Denv2C (Figure M2A). The real-time increase in Cy3 fluorescence traces of Denv2C-promoted 5UAR strand displacement from 5UAR/c5UAR annealed duplexes were fitted to equation [2]. I(t) is the actual fluorescence intensity at 560 nm, upon excitation at 532 nm, I_0_ and I_f_ are the fluorescence intensities of Cy3/atto647N-labelled 5UAR/c5UAR before and after completion of strand displacement reaction kinetics, respectively. The parameter t_0_ is the start time of the reaction. The fitted pseudo first order reaction rate for strand transfer reactions is k_obs’_. Like annealing reactions, the strand transfer reaction rates, k_obs’_, were plotted against the concentration of non-labelled 5UAR (Figure M2B) and were observed to linearly vary with increasing concentrations of 5UAR-nl. As mentioned earlier, the linear relationship between k_obs’_ and [5UAR-nl] follows equation [3] [6, 7, 9] and based on the acquired kinetic parameters coupled to the continuous increase in donor intensity plateau (Figure M2A), we propose the following reaction mechanism [4]:


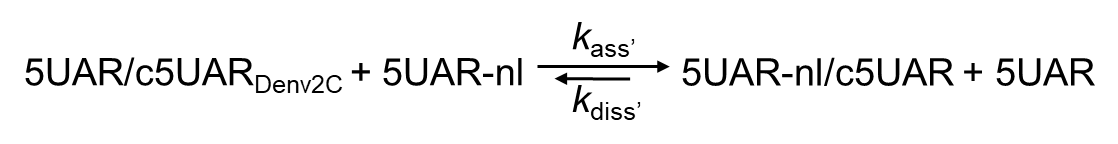
 Scheme 3

where the final displaced duplex, 5UAR-nl/c5UAR, is formed through a bimolecular reaction [5-7] and is governed by the second order association constant, k_ass’_, and the first order dissociation constant, k_diss’_, respectively. The obtained values of kinetic parameters are provided in Table M2.

**
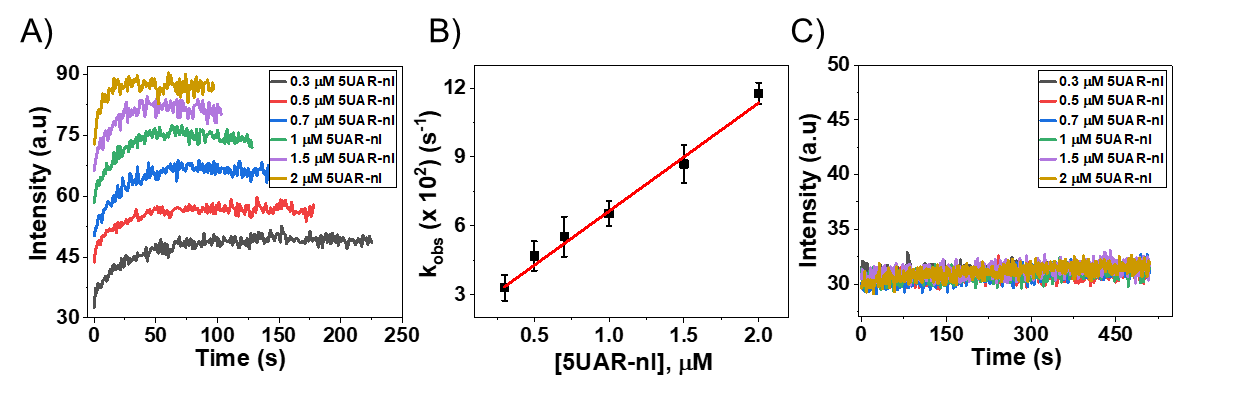
**

**Figure M2:** Real time progress curves and kinetic parameters of 5UAR-nl/c5UAR strand displacement in presence of Denv2C (A and B) and Denv2C^S34C^ (C). Progress curves of 5UAR-nl/c5UAR strand displacement were initiated from the duplex formed during 20 nM Cy3-lablled 5UAR and 20 nM Atto647N-labelled c5UAR annealing as described in figure 2, by adding non-labelled 5UAR (5UAR-nl) in at least 10-fold higher concentrations (concentration shown in legend) in the presence of 2 µM concentration of either (A) Denv2C or (C) Denv2C^S34C^. To accelerate the reaction kinetics, all strand displacement reactions were performed at 37˚C. Excitation and emission wavelengths were 532 nm and 560 nm, respectively. Denv2C-assisted 5UAR-nl/c5UAR strand displacement traces were fitted using equation (2). Obtained apparent rates (B) k_obs_, in the presence of Denv2C were plotted against increasing concentrations of the complementary non-labelled 5UAR and fitted using equation (3) and values are provided in Table M2. No strand displacement was observed in the presence of Denv2C^S34C^.

By contrast, we observed no changes in the Cy3 fluorescence of the Cy3/atto647N-labelled 5UAR/c5UAR (Figure M2C) duplexes in the presence of Denv2C^S34C^, thus suggesting that no strand displacement takes place. We tested up to 100-fold molar excess 5UAR-nl to monitor strand-displacement but could not detect any increase in Cy3 fluorescence showing the inability of Denv2C^S34C^ to initiate strand displacement at even high concentrations of complementary strands.

**Table M2:** **Kinetic parameters of 5UAR-nl/c5UAR strand displacement in the absence and presence of either Denv2C or Denv2C^S34C^.** Kinetic rate constants, k_ass’_ and k_diss’_ were calculated from the dependence of the k_obs_ values on the concentration of the non-labelled 5UAR, using equation [3] as indicated in Figure 4. Strand displacement reactions were performed in pseudo-first order conditions by adding 5UAR-nl at least 10-fold molar excess of the 5UAR/c5UAR annealed duplex.

| **Cy3/Atto647N-labelled**  **ORN**  **Duplex** | **Non-labelled**  **strand displacement**  **ORN** | **Protein**  **(2 µM)** | ***k*_ass’_ (M^−1^s^−1^)×10^−3^** | ***k*_diss’_ (s^−1^)×10^4^** |
| --- | --- | --- | --- | --- |
| 5UAR/c5UAR | 5UAR-nl | **-** | No strand Displacement | |
|  |  | Denv2C | 47 (±3) | 193 (±35) |
|  |  | Denv2C^S34C^ | No Strand Displacement | |


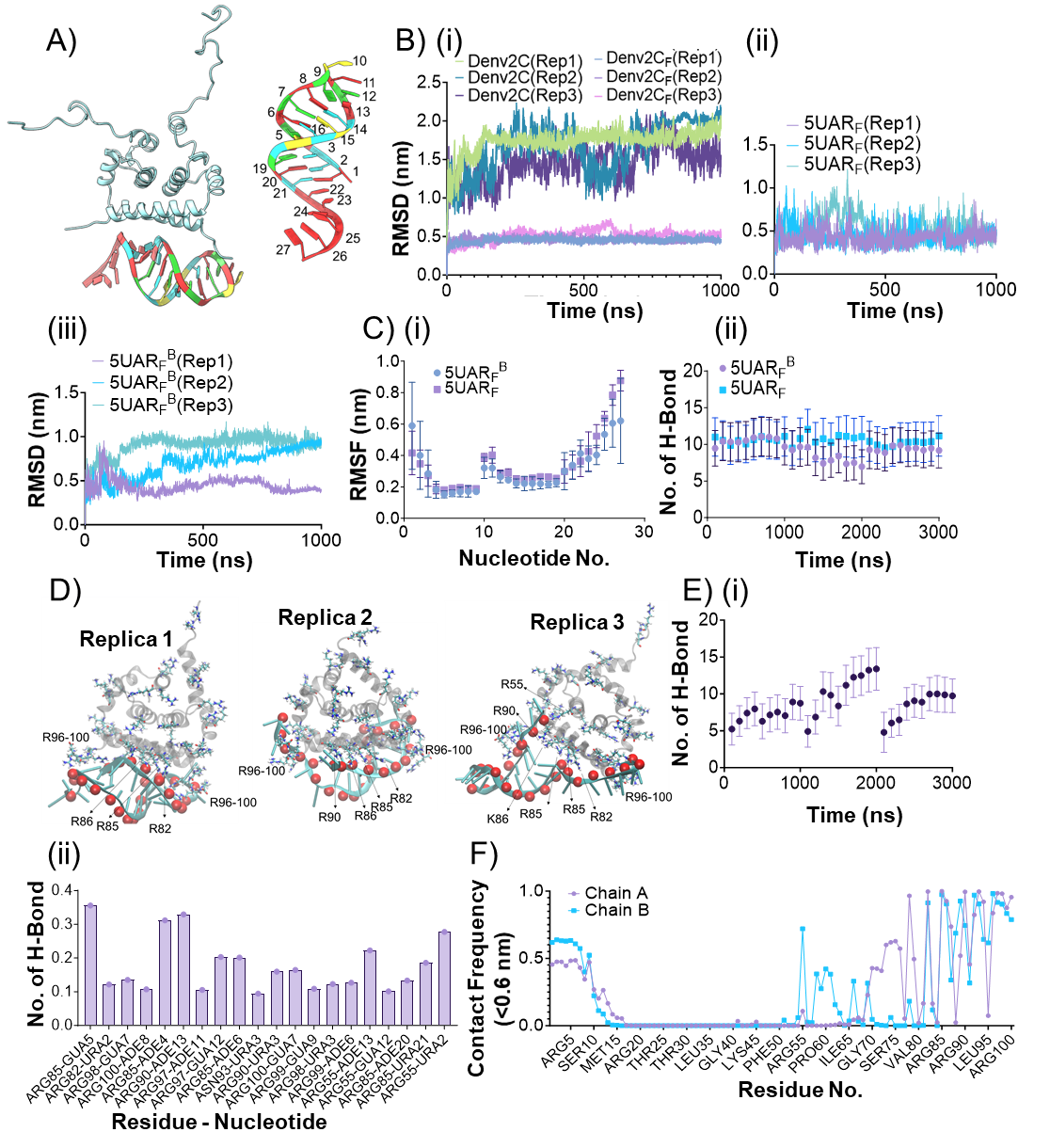


**Figure S4:** MD simulations reveal Denv2C:5UAR_F_ structural dynamics and interactions (A) Cartoon representation of starting frame of 5UAR_F_ in the presence (5UAR_F_^B^) and absence of Denv2C(5UAR_F_). (B) Root mean square deviation (RMSD) of (i) full-length (Denv2C) and folded region (Denv2C_F_, residue 20-100 aa), 5UAR_F_ in the (ii) presence and (iii) absence of Denv2C. (C) (i) RMSF per nucleotide of 5UAR_F_ in absence (5UAR_F_) and presence (5UAR_F_^B^) of Denv2C. (ii) Block average showing inter-5UAR_F_ hydrogen bonds (H-bond) at every 50 ns in the absence (5UAR_F_) and presence (5UAR_F_^B^) of Denv2C. (D) Cartoon representation of final frames taken from 5UAR_F_:Denv2C complex from three different replicates. The folded region of Denv2C is shown as gray transparent cartoon with ARG and LYS residues represented as sticks while the RNA phosphate backbone atoms are shown as red spheres in each replica. (E) (i) Block averaged number of hydrogen bonds between Denv2C and 5UAR_F_ at every 100 ns and (ii) the hydrogen bond lifetime between Denv2C residues and 5UAR_F_ nucleotides are shown from three different replicates. H-bond lifetimes greater than 10% of 3000 ns long simulation time i.e., 0.1 are shown. (F) Per-residue contact fraction of Denv2C with 5UAR_F_ based on the 0.6 nm cutoff distance.


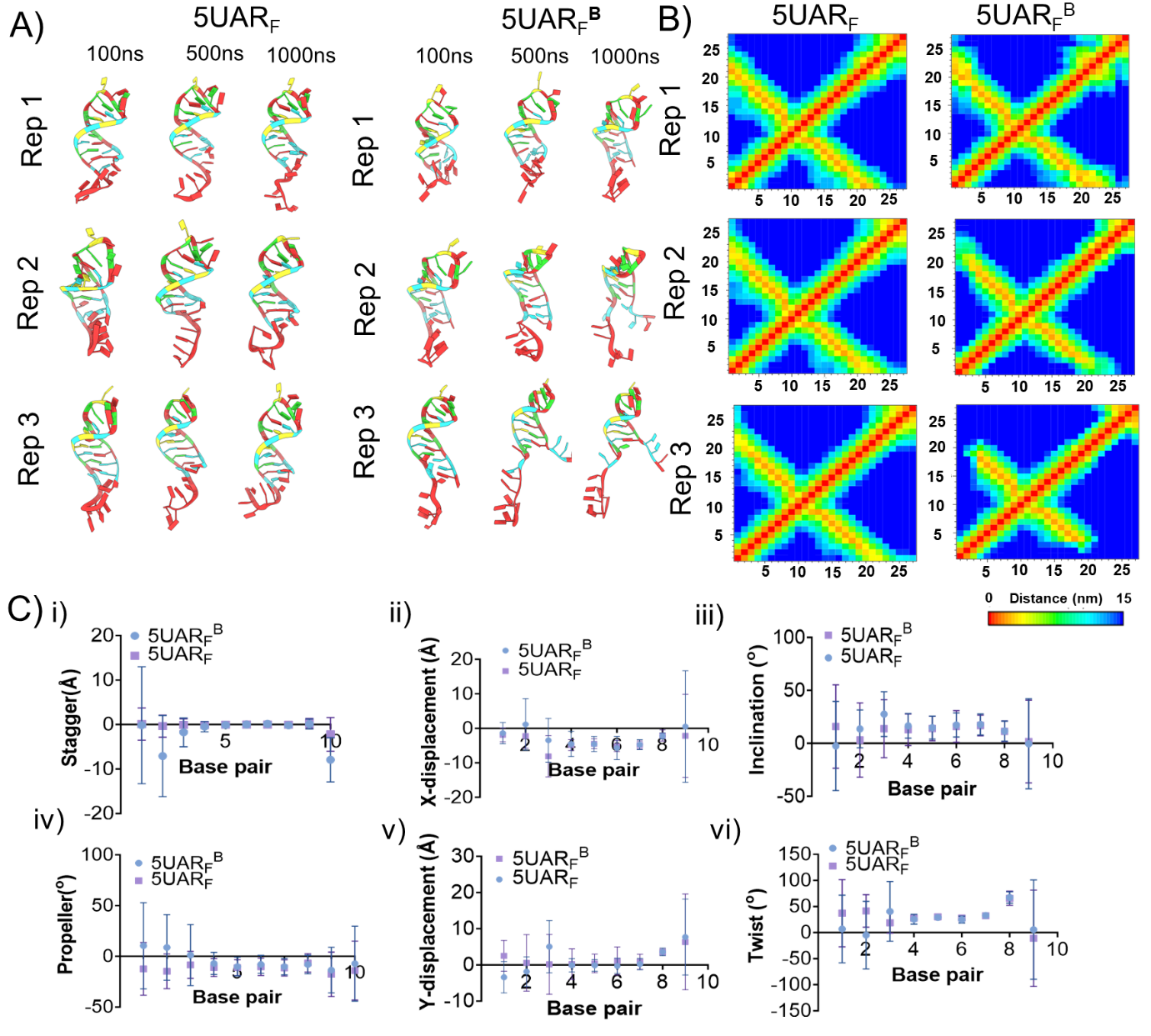


**Figure S5:** Effect of Denv2C binding on 5UAR_F_. (A) Snapshots of 5UAR_F_ in the absence (5UAR_F_) and presence (5UAR_F_^B^) of Denv2C at 100 ns, 500 ns and 1000ns from 1µs long simulation trajectory. (B) Time average contact maps for 5UAR_F_ in the absence (5UAR_F_) and presence (Denv2C:5UAR_F_) of Denv2C from three replicate simulations. (C) Time averaged base pair (Stagger and Propeller) and base step (X/Y-displacement, Inclination, Twist) parameters from combined trajectories of three independent 500 ns long 5UAR_F_ simulations in the absence (Free_5UAR_F_) and presence (Bound_5UAR_F_) of Denv2C. (Base pairs ids: 1:1=21, 2:2=20, 3:3=19, 4:4=18, 5:5=17, 6:6=16, 7:7=15, 8:8=14, 9:9=13, 10:24=25; Base step ids: 1:1=21/2=20, 2:2=20/3=19, 3:3=19/4=18, 4:4=18/5=17, 5:5=17/6=16, 6:6=16/7=15, 7:7=15/8=14, 8:8=14/9=13, 9:9=13/24=25).


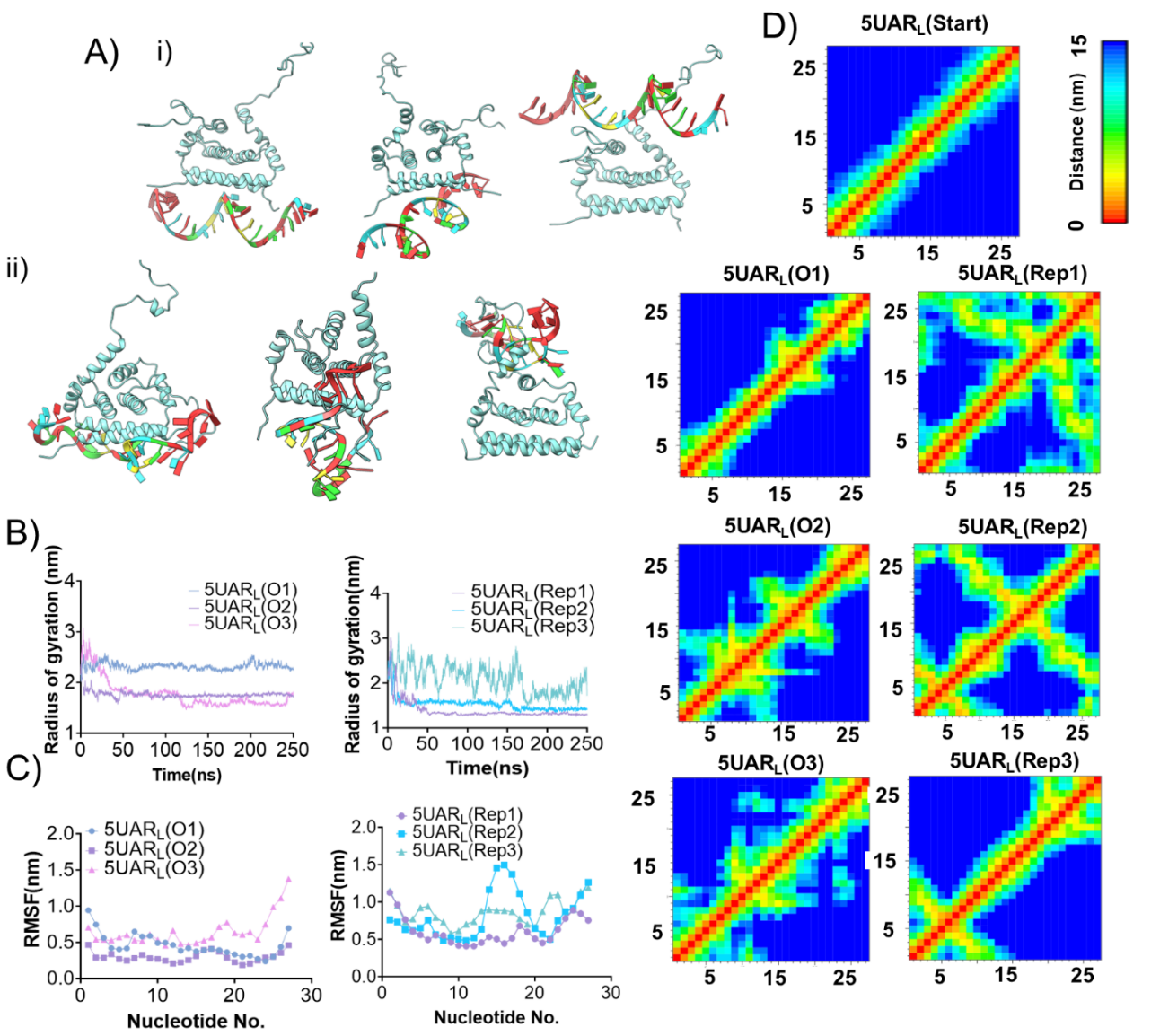


**
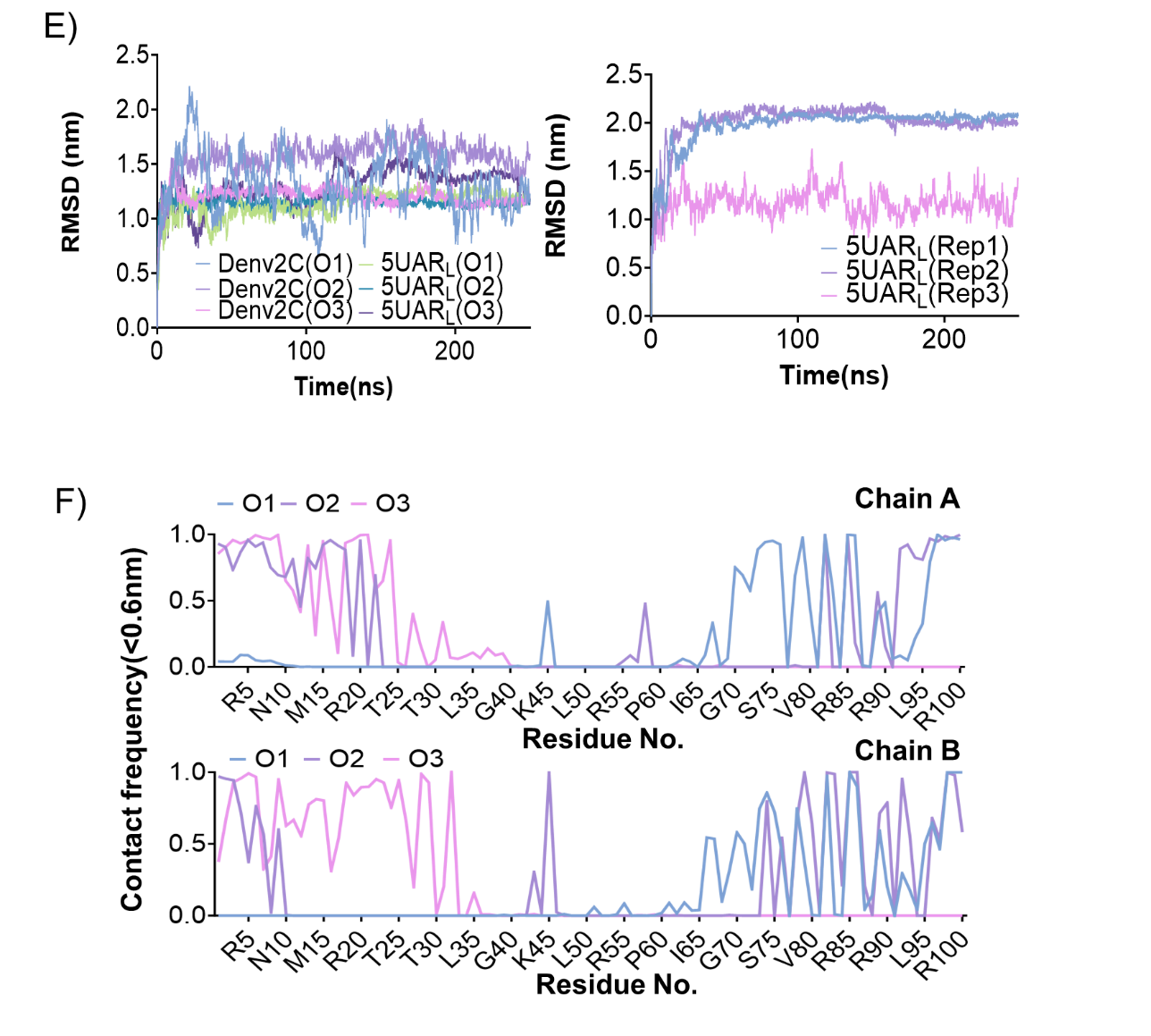
**

**Figure S6:** MD simulations showing binding of Denv2C with the 5UAR hairpin in either linearized or folded conformation. (A-B) Cartoon representation of starting and final frame of the Denv2C in the presence of the 5UAR molecule in three different orientations, O1: 5UAR parallel to α4-helix, O2: 5UAR proximal to α1-helix and O3: 5UAR perpendicular to α4-helix. (C) Plot showing radius of gyration of (i) the Denv2C-bound 5UAR and (ii) the linearized 5UAR in different orientation. (D) Per nucleotide root mean squared fluctuation (RMSF) values of (i) the Denv2C-bound-5UAR and (ii) the linearized 5UAR. All values are obtained from three different 250 ns trajectories. (E) Time average contact maps of the Denv2C-bound-5UAR and the linearized 5UAR in three orientations (O1 to O3). All simulations were performed in triplicates (Rep1-Rep3). (E) RMSD over the simulation time of Denv2C protein backbone atoms and 5UAR_L_ in complex (left) and free 5UAR_L_ (right) MD simulation from three independent 250 ns long simulation trajectory. (F) Per-residue contact fraction between chain A/B from Denv2C with 5UAR_L_ based on the 0.6 nm cutoff distance from each of the three simulations with different orientations of 5UAR_L_.


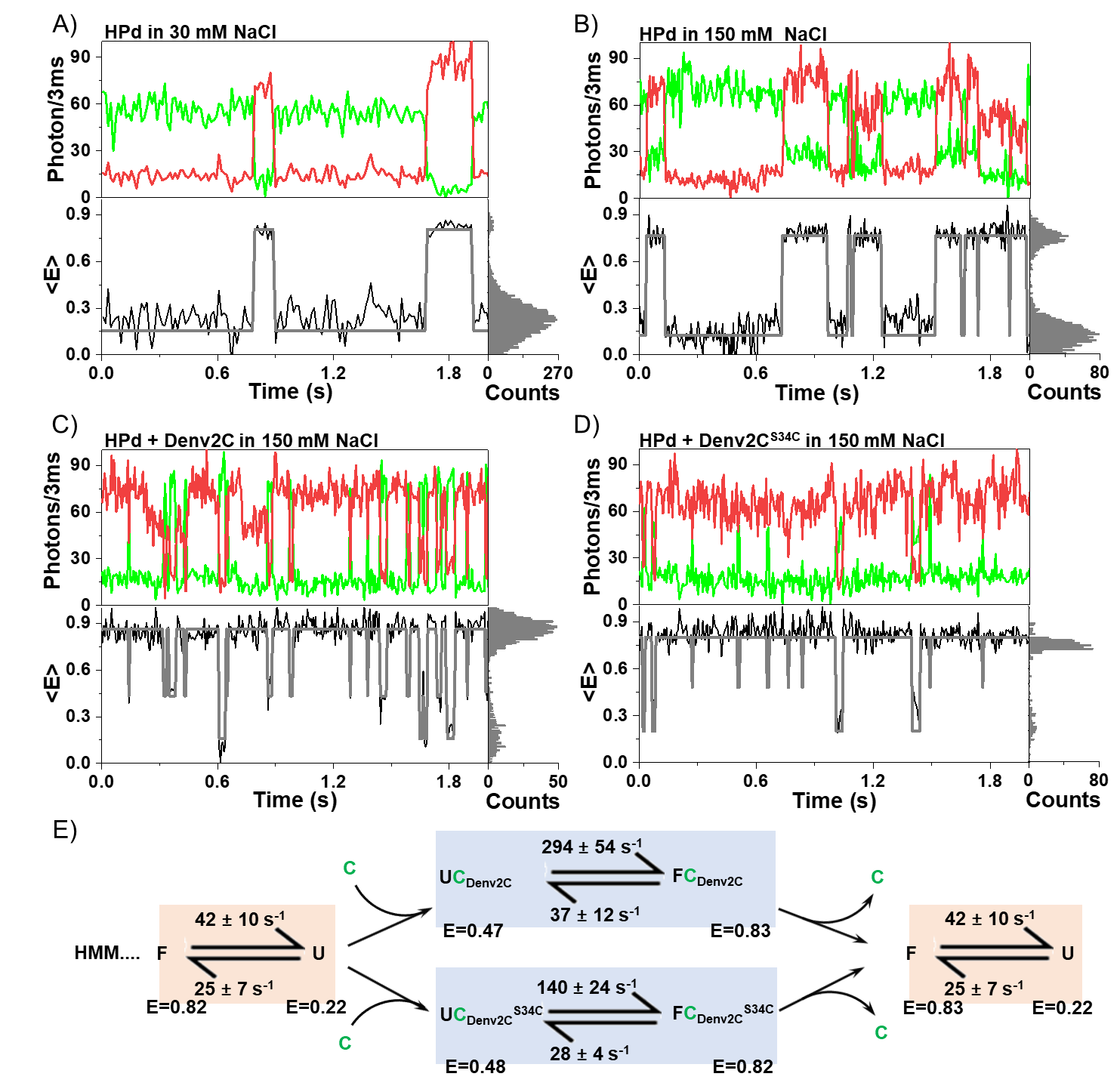


**Figure S7:** Chaperone-accelerated DNA based hairpin (HPd) folding affected by structured domain. Representative donor (green) and acceptor (red) fluorescence time traces depicting unassisted folding of the surface-immobilized 5′-3′ FRET-labelled DNA hairpin (HPd) in the presence of (A) 30 mM NaCl and (B) 150 mM NaCl. The uncorrected transfer efficiency, <E>, (black) and the most likely state trajectory (gray) based on the hidden markov model (HMM) are shown in below panel. Analogous to (B), but depicting (C) Denv2C-assisted and (D) Denv2C^S34C^-assisted HPd folding with saturating concentrations of protein (200 nM) to ensure that HPd molecules are ways chaperone-associated. (E) Kinetic 4-state model for Denv2C and its mutant-assisted folding. The folded, F, and unfolded, U, conformations of the donor-acceptor labelled HPd freely interconvert in the absence of chaperone protein, C, with an equilibrium constant that favors U. When the chaperone protein is bound, the unfolded, UC (UC_Denv2C_ and UC_Denv2C_^S34C^), and folded, FC (FC_Denv2C_ and FC_Denv2C_^S34C^), conformations of the hairpin still interconvert, but with an equilibrium constant that favors F. However, that favorable equilibrium towards F conformation reduced by ~2.1-fold during Denv2C^S34C^-assisted folding as compared to Denv2C-assisted folding of HPd. All obtained values of transfer efficiency (<E>) and associated transition rates (k) are reported in table 2.

**
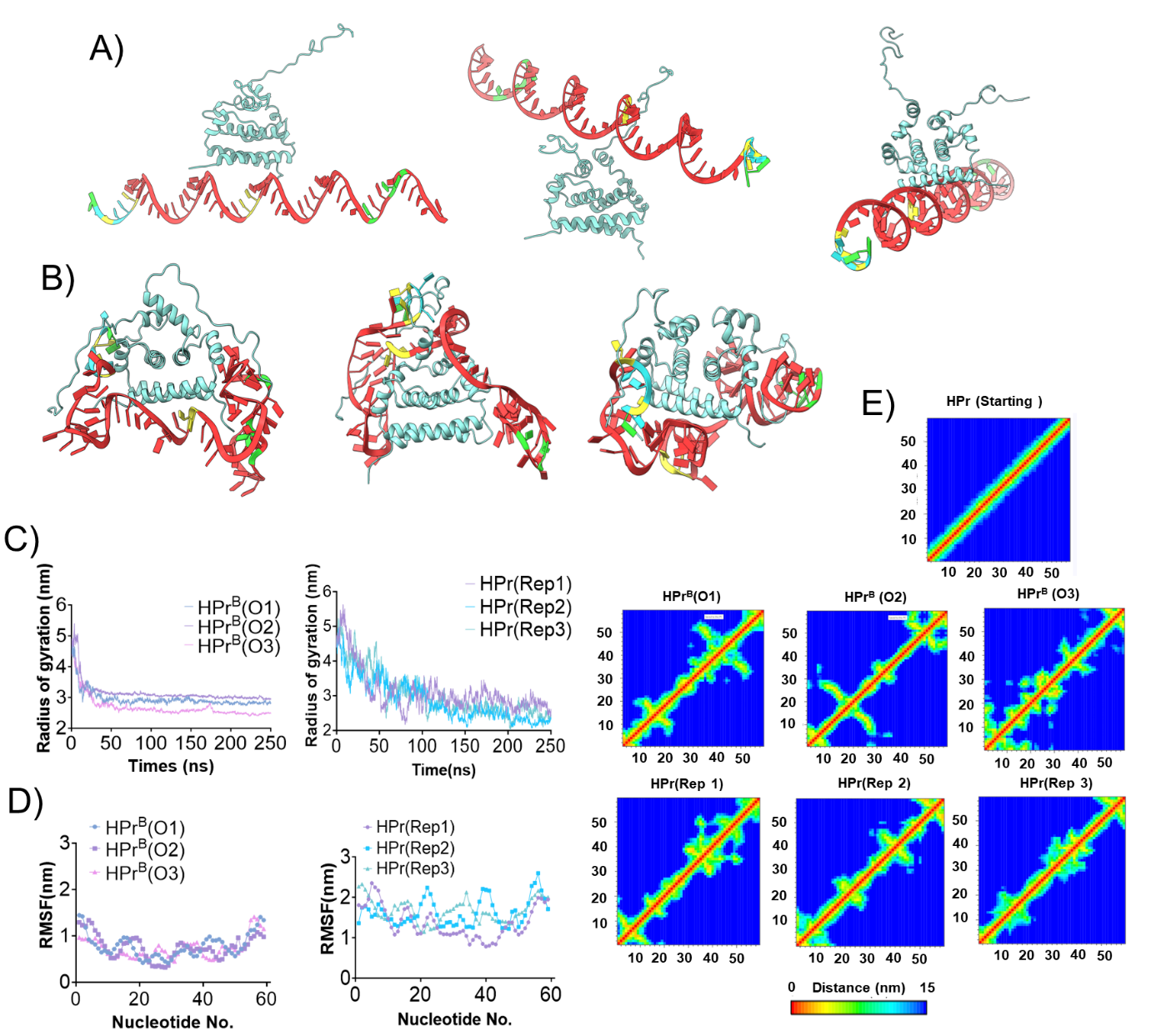
**

**
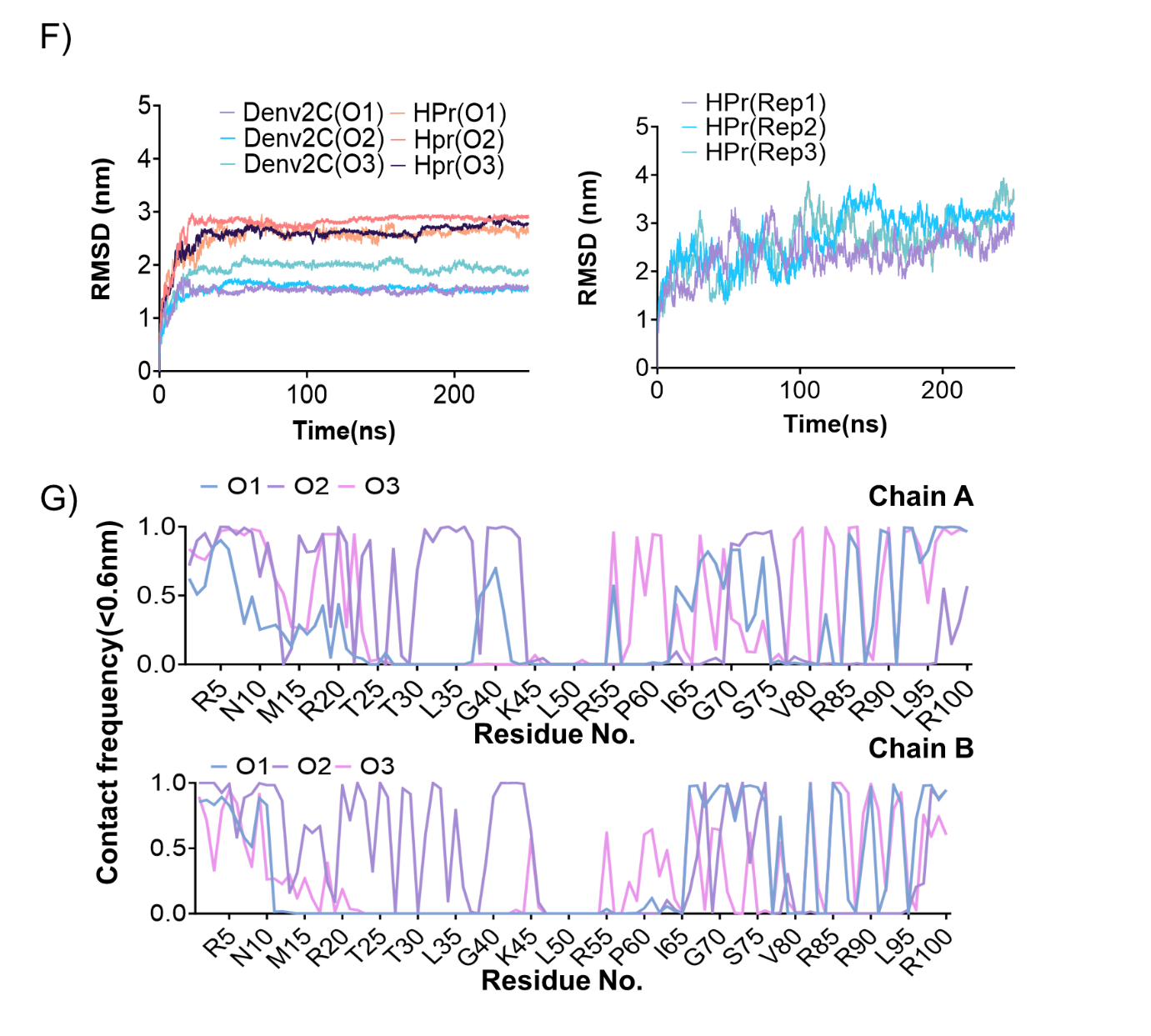
**

**Figure S8:** Denv2C mediated HPr condensation. (A-B) Cartoon representation of first and final frame of C protein in the presence of the HPr molecule in three different orientation (O1: HPr parallel to α4-helix, O2: HPr proximal to α1-helix and O3: HPr perpendicular to α4-helix). (C) HPr radius of gyration over the simulation timer and (D) per-nucleotide root mean squared fluctuation (RMSF) of the HPr molecule in the absence (top) and in the presence (bottom) of Denv2C. Three replicates (Rep1-Rep3) from three different 250 ns trajectories are shown for comparison. (E) Mean distance contact maps of HPr bound to C protein and free 5UAR simulations in orientation O1 to O3 and three replicate simulations of each 250ns long compared to the contact map starting state of HPr. (E) RMSD over the simulation time of Denv2C protein backbone atoms and HPr in complex (left) and free HPr (right) MD simulation from three independent 250 ns long simulation trajectory. (F) Per-residue contact fraction between chain A/B from Denv2C with HPr based on the 0.6 nm cutoff distance from each of the three simulations with different orientations of HPr.


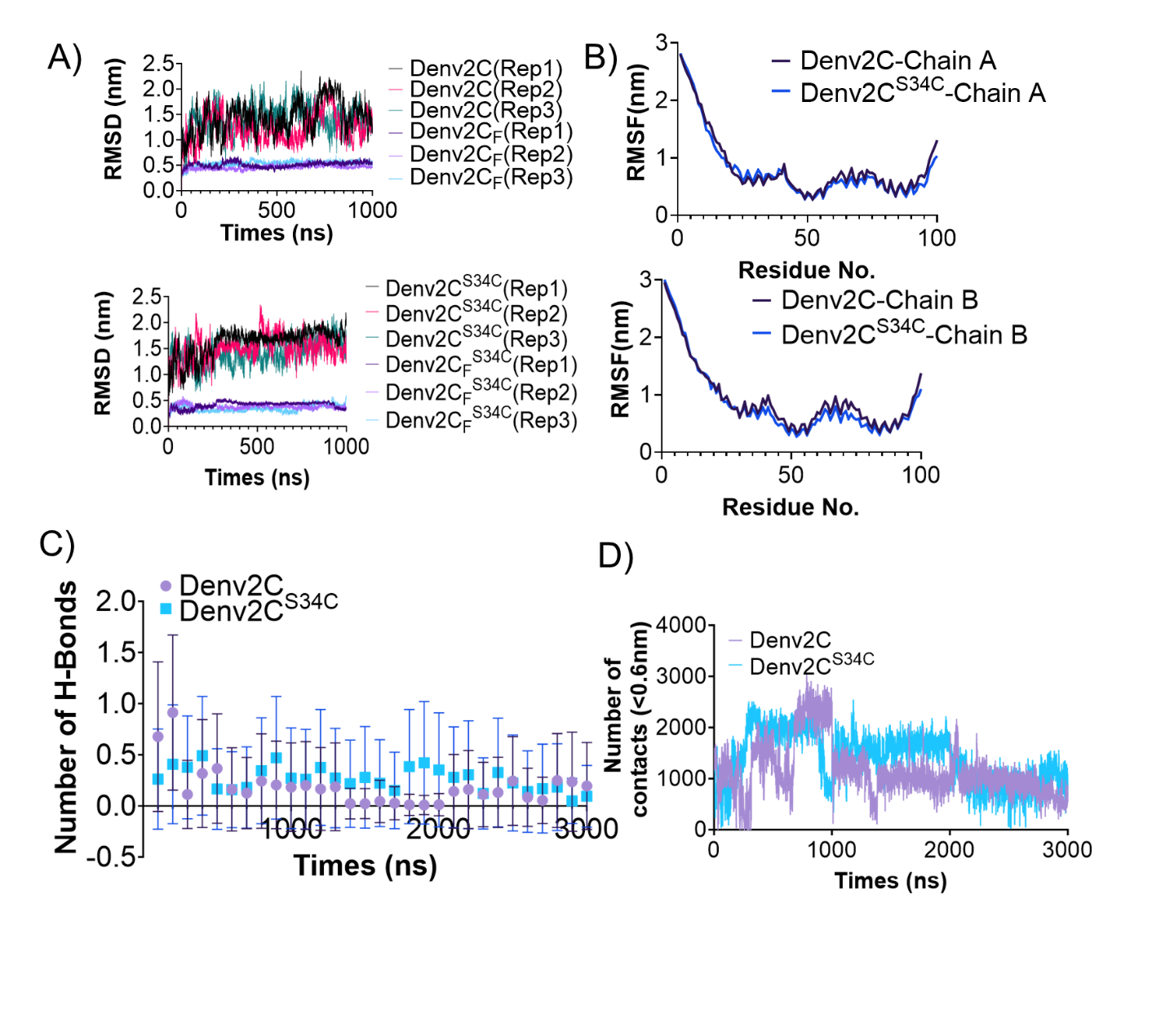

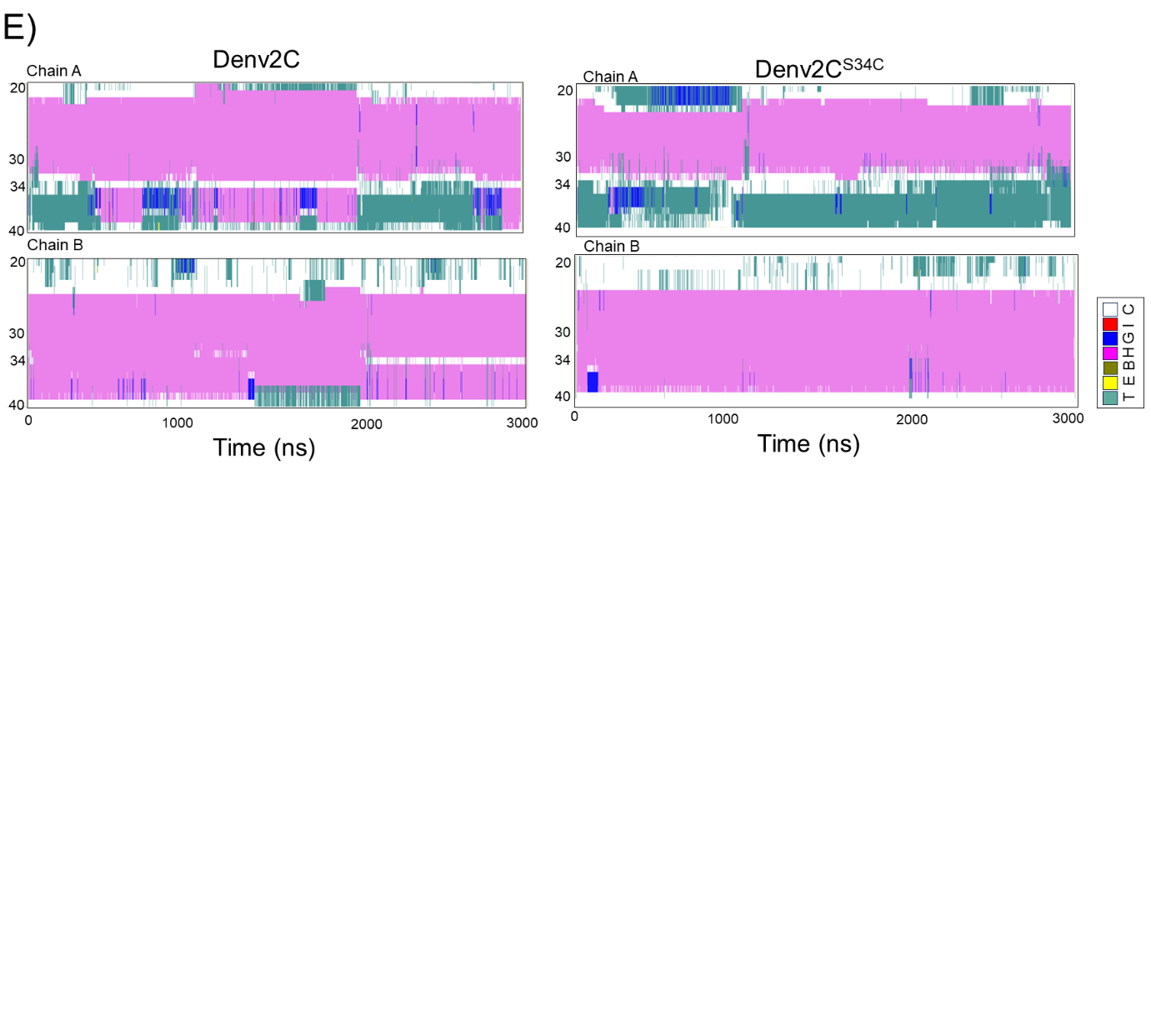


**Figure S9:** **Denv2C and Denv2C^S34C^ intrinsic dynamics.** **A)** Root mean square deviation (RMSD) of protein backbone atoms of full-length (residues 1-100) of Denv2C and Denv2C^S34C^, and for core fold regions only (residues 20-100, Denv2C_F_ and Denv2C_F_^S34C^, each for three replicate 1000 ns simulations (Rep1, Rep2, Rep3). B) Per-residue root mean square fluctuations (RMSFs) of each monomer of Denv2C and Denv2C^S34C^ for combined triplicate 1000 ns simulation trajectories. **C)** Block average of number of hydrogen bonds between α1 helices (residues 20-40) of each monomer at every 100 ns from Denv2C and Denv2C^S34C^ triplicate 1000 ns simulations. **D)** Plot showing the number of contacts between α1 helices (residues 20-40) of each monomer from Denv2C and Denv2C^S34C^ triplicate 1000 ns simulations. **E)** Per-residue secondary structure changes of region spanning residues 20-40 from each monomer of Denv2C and Denv2C^S34C^ based on combined MD simulation trajectories from three replicate 1000 ns simulations. Secondary structures are color-coded as per key (T: Turn, E: Elongated, B: Beta sheet, H: α,-helix, G: 3_10_-helix, I:5-helix, C:Coil).


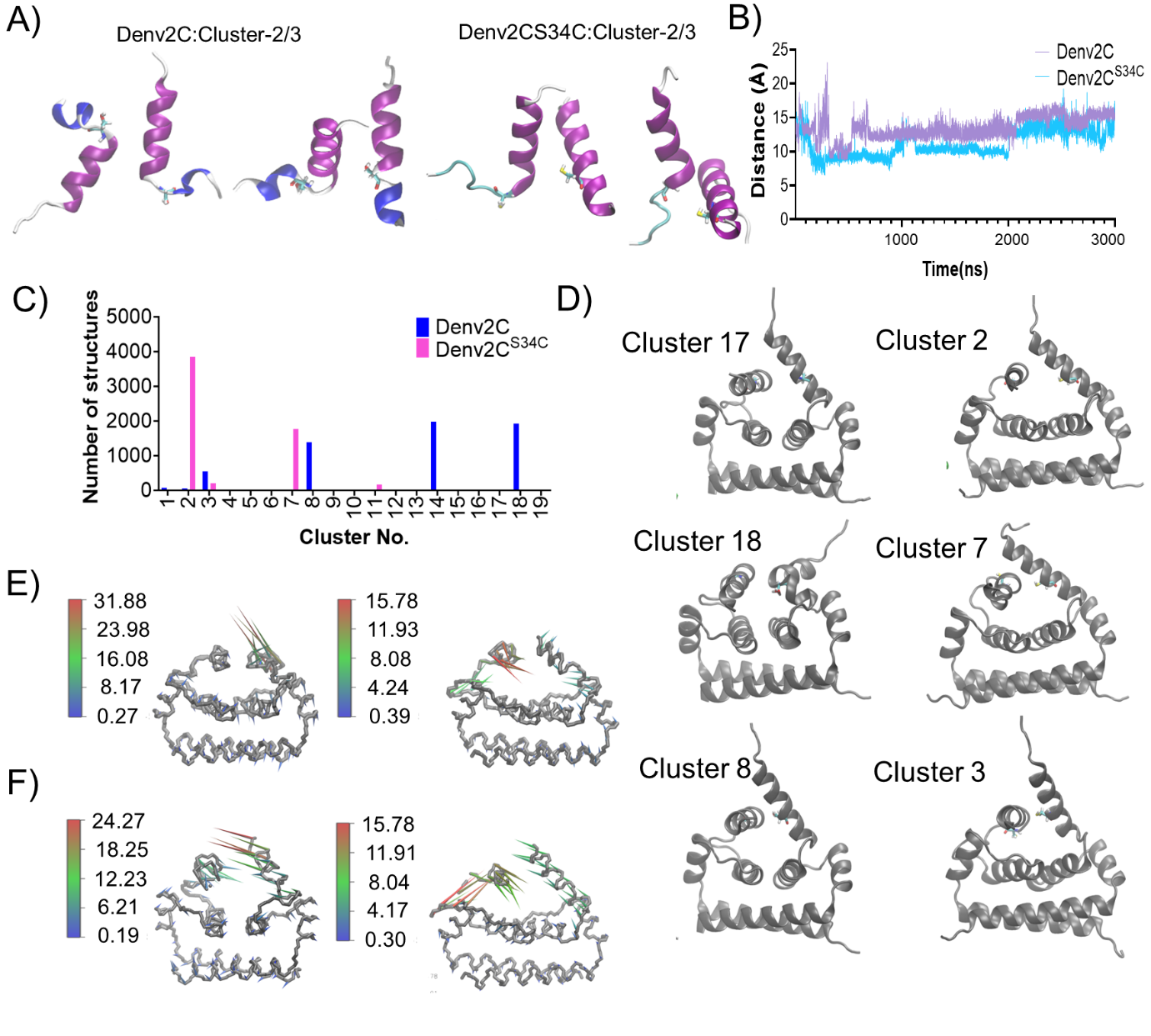


**Figure S10:** **MD simulation reveals differences in Denv2C and Denv2C^S34C^ protein dynamics. A)** Representative structures of α1-helices from second and third most populated clusters identified from 3000 ns long combined simulation trajectory of Denv2C and Denv2C^S34C^.**B)** Plot showing the distance between Thr_30_ from each monomer of Denv2C and Denv2CS34C dimers with respect to combined triplicate simulation trajectory time of 3000ns. **C-D)** Bar plots showing the number of structures in each cluster from Denv2C and Denv2C^S34C^ along with representative structures of folded region (Denv2C(left) and Denv2C^S34C^(right)) from three most populated clusters. Cluster analysis was performed on combined trajectories from three replicate 1000 ns simulations each for Denv2C and Denv2C^S34C^. **(E)** Denv2C and **(F)** Denv2C^S34C^, showing first and second principal components (PC1 (left) and PC2 (right)) capturing the dominant motions from 3000 ns long combined simulation trajectories from three 1000 ns replicate simulations.


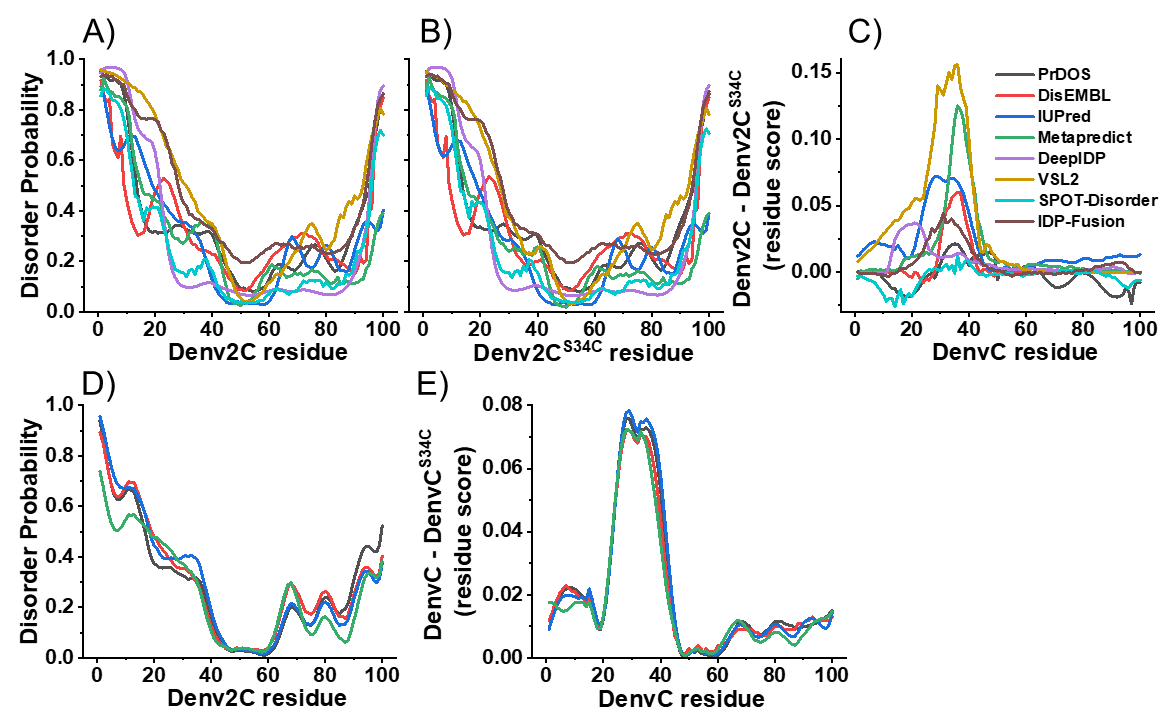


**Figure S11: Per-residue disorder profile for DenvC protein and effect of S34C mutation.** Predicted disorder propensity of the (A) Denv2C and (B) Denv2C^S34C^, based on primary amino acid sequences. Disorder scores were computed using multiple prediction algorithms via the CAID portal. Residue scores above 0.5 indicate a high probability of intrinsic disorder. (C) **Structural impact of the S34C mutation.** Residue-wise difference scores (Residue score) were calculated by subtracting the predicted disorder values of Denv2C^S34C^ from the native Denv2C. Positive values indicate regions with a mutation-induced disorder-to-order transition. (D) Comparative disorder profiles predicted by IUPred for Denv2C (black trace) and capsid proteins from other serotypes: Denv1C (Myanmar 49440 strain; gray), Denv3C (05K863DK1 strain; blue), and Denv4C (06K2270DK1 strain; green). (E) **Effect of S34C substitution across dengue serotypes.** Using IUPred prediction algorithm, the serine at position 34 was computationally replaced with cysteine in Denv1C (Denv1C^S34C^) (gray trace), Denv3C (Denv3C^S34C^) (blue trace) and Denv4C (Denv4C^S34C^) (green trace). Residue-wise difference scores were computed by subtracting the mutant disorder values from their respective wild-type sequences (Denv1C: gray trace, Denv3C: blue trace, Denv4C: green trace) and compared to that of Denv2C (black trace). These sequence-based predictions suggest that ordered regions remain structurally conserved irrespective of dimerization, while residue 34 plays a key role in modulating local disorder. All predictions were performed using the CAID Prediction Portal (https://caid.idpcentral.org), which integrates 33 state-of-the-art disorder prediction algorithms.

**Transient binding of Denv2C and Denv2C^S34C^**

The RNA secondary structure is stabilized through the cyclic RNA binding and release of the RNA chaperone (transient binding time), coupled with a reciprocal entropy transfer process between the RNA and the protein. Therefore, Denv2C interaction is needed for the disorder-to-order rearrangement of the RNA but not to maintain its structure. On the other hand, an annealer like Denv2C^S34C^ enhances the local concentration of protein by binding to RNAs for longer duration to increase the probability of RNA-RNA interactions. Therefore, the transient binding time of the RNA chaperone during its RNA interaction should be smaller as compared to an annealer. Hence, we obtained the transient binding time of Denv2C and Denv2C^S34C^ on the ubiquitous RNA hairpin (HPr). Our smFRET results showed that the addition of either Denv2C or Denv2C^S34C^ favors the folded conformation of the HPr with FRET value of <E> = ~0.8 as well as increase the probability of finding HPr in that folded state. Therefore, HPr’s ability to remain in folded conformation and subsequent reduced interconversion rate (k_F🡪U_) from <E> = ~0.8 to <E> = ~0.2 can directly be proportional to the transient binding time of the attached protein on HPr hairpin. We observed that the interconversion rate of HPr unfolding (k_F🡪U_) decreased by ~10-folds and ~20-folds in the presence of Denv2C and Denv2C^S34C^, respectively, suggesting Denv2C^S34C^ transiently binds HPr for extended time as compared to Denv2C.

**
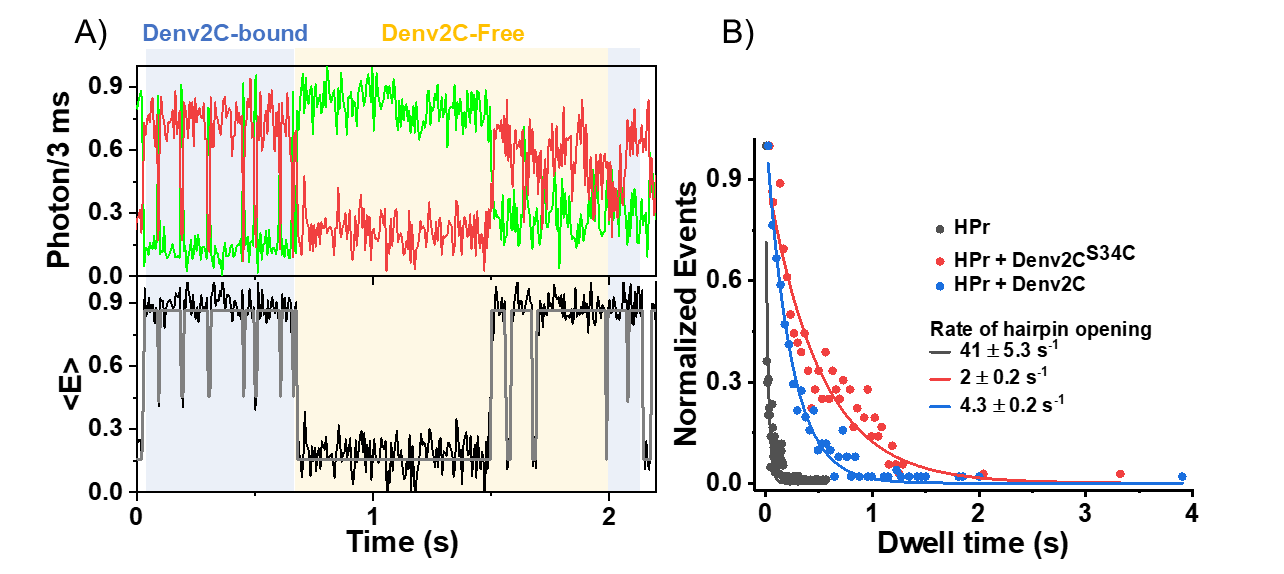
**

**Figure S12:** Folding transitions of RNA based hairpin (HPr) indicating cycles of chaperone transient binding time. (A) Representative donor (green) and acceptor (red) fluorescence time traces depicting folding transitions of the surface-immobilized 5′-3′ FRET-labelled RNA hairpin (HPr) in Denv2C-bound (blue box) and Denv2C-free conditions. The uncorrected transfer efficiency, <E>, (black) and the most likely state trajectory (gray) based on the hidden markov model (HMM) are shown in below panel. (B) Dwell time showing the unfolding kinetics of HPr in the absence (black dots) and presence of either 200 nM Denv2C (blue dots) or 200 nM Denv2C^S34C^ (red dots). The dwell time values were fitted using exponential equation. The interconversion rate of the donor-acceptor labelled HPr from unfavorable F conformation (<E> = ~0.85) to favorable U conformation (<E> = ~0.2) drastically decreases in the presence of Denv2C (by ~10-folds) and Denv2C^S34C^ (by ~20-folds) suggesting Denv2C^S34C^ transiently binds longer on HPr when compared to Denv2C.

**References**

E. D. Holmstrom, Z. Liu, D. Nettels, R. B. Best and B. Schuler Disordered RNA chaperones can enhance nucleic acid folding via local charge screening **Nat Commun** *10* (2019) 2453. DOI: 10.1038/s41467-019-10356-0.

S. V. Avilov, J. Godet, E. Piemont and Y. Mely Site-specific characterization of HIV-1 nucleocapsid protein binding to oligonucleotides with two binding sites **Biochemistry** *48* (2009) 2422-2430. DOI: 10.1021/bi8022366.

T. M. Nordlund, S. Andersson, L. Nilsson, R. Rigler, A. Graslund and L. W. McLaughlin Structure and dynamics of a fluorescent DNA oligomer containing the EcoRI recognition sequence: fluorescence, molecular dynamics, and NMR studies **Biochemistry** *28* (1989) 9095-9103. DOI: 10.1021/bi00449a021.

X. E. Yong, V. R. Palur, G. S. Anand, T. Wohland and K. K. Sharma Dengue virus 2 capsid protein chaperones the strand displacement of 5'-3' cyclization sequences **Nucleic Acids Res** *49* (2021) 5832-5844. DOI: 10.1093/nar/gkab379.

C. Boudier, R. Storchak, K. K. Sharma, P. Didier, A. Follenius-Wund, S. Muller, J. L. Darlix and Y. Mely The mechanism of HIV-1 Tat-directed nucleic acid annealing supports its role in reverse transcription **J Mol Biol** *400* (2010) 487-501. DOI: 10.1016/j.jmb.2010.05.033.

K. Sharma, P. Didier, J. L. Darlix, H. de Rocquigny, H. Bensikaddour, J. P. Lavergne, F. Penin, J. M. Lessinger and Y. Mely Kinetic analysis of the nucleic acid chaperone activity of the hepatitis C virus core protein **Nucleic Acids Res** *38* (2010) 3632-3642. DOI: 10.1093/nar/gkq094.

K. K. Sharma, H. de Rocquigny, J. L. Darlix, J. P. Lavergne, F. Penin, J. M. Lessinger and Y. Mely Analysis of the RNA chaperoning activity of the hepatitis C virus core protein on the conserved 3'X region of the viral genome **Nucleic Acids Res** *40* (2012) 2540-2553. DOI: 10.1093/nar/gkr1140.

X. E. Yong, P. V. Raghuvamsi, G. S. Anand, T. Wohland and K. K. Sharma Dengue virus strain 2 capsid protein switches the annealing pathway and reduces intrinsic dynamics of the conserved 5' untranslated region **RNA Biol** *18* (2021) 718-731. DOI: 10.1080/15476286.2020.1860581.

J. Godet, C. Boudier, N. Humbert, R. Ivanyi-Nagy, J. L. Darlix and Y. Mely Comparative nucleic acid chaperone properties of the nucleocapsid protein NCp7 and Tat protein of HIV-1 **Virus Res** *169* (2012) 349-360. DOI: 10.1016/j.virusres.2012.06.021.
